# Semaglutide use linked to lower COVID-19 and influenza severity with better renal outcomes after pandemic or seasonal infection

**DOI:** 10.1093/biomethods/bpag042

**Published:** 2026-07-17

**Authors:** Robert Matson, A J Venkatakrishnan, Karthik Murugadoss, Venky Soundararajan

**Affiliations:** nference, 1 Main Street, East Arcade Building, Cambridge, MA 02142, United States; nference, 1 Main Street, East Arcade Building, Cambridge, MA 02142, United States; Metabolism Agentic Intelligence Atlas (MAIA), Cambridge, MA, Cambridge, MA 02142, United States; nference, 1 Main Street, East Arcade Building, Cambridge, MA 02142, United States; Metabolism Agentic Intelligence Atlas (MAIA), Cambridge, MA, Cambridge, MA 02142, United States; nference, 1 Main Street, East Arcade Building, Cambridge, MA 02142, United States; Metabolism Agentic Intelligence Atlas (MAIA), Cambridge, MA, Cambridge, MA 02142, United States

**Keywords:** COVID, flu, semaglutide, GLP-1, unvaccinated, kidney disease, acute kidney injury, incretins

## Abstract

Respiratory viral infections continue to impose a large burden of hospitalization, organ failure and death, particularly among older adults and people with cardiometabolic disease. Whether prior semaglutide exposure is associated with lower post-infection severity across distinct respiratory viruses remains uncertain. We analyzed de-identified longitudinal electronic health record data from a federated U.S. network spanning more than 29 million patients. Adults with documented COVID-19 or influenza infection were classified by pre-infection semaglutide use versus metformin use without prior GLP-1 receptor agonist exposure and one-to-one propensity matched on demographics, BMI, HbA1c, vaccination status, prior infection status, antiviral use, healthcare utilization extent, and baseline comorbidities, yielding matched cohorts of *N* = 7092 for COVID-19 and *N* = 1920 for influenza (flu). In the COVID-19 cohort, semaglutide exposure was associated with significantly lower 30-day mortality (0.3% vs 0.8%, RR 0.39, *P* < .001), hospitalization (5.3% vs 7.4%, RR 0.72, *P* < .001), and the composite disease severity outcome (8.2% vs 10.6%, RR 0.77, *P* < .001). Differences in rates of intensive care admission (1.4% vs 1.8%, RR 0.79, *P* = .07) and mechanical ventilation (0.7% vs 0.9%, RR 0.76, *P* = .14) were not statistically significant. In the flu cohort, semaglutide use was also associated with significantly lower mortality (0.2% vs 0.7%, RR 0.29, *P* = .02), hospitalization (6.5% vs 9.6%, RR 0.68, *P* < .001), and the composite outcome (10.3% vs 14.6%, RR 0.70, *P* < .001); ICU admissions (1.9% vs 2.7%, RR 0.71, *P* = .11) and mechanical ventilation (0.7% vs 0.9%, RR 0.78, *P* = .48) were not statistically significant. Among organ-specific outcomes, for which *P* values were adjusted for multiple comparisons, a significant risk reduction was observed for acute kidney injury (COVID-19 RR 0.73, *P* = .02; flu RR 0.58, *P* = .03) in the 30-day observation period, whereas reductions in acute coronary syndrome (COVID-19 RR 0.67, *P* = .14; flu RR 1.00, *P* = 1.00) and acute respiratory failure (COVID-19 RR 0.84, *P* = .17; flu RR 0.74, *P* = .27) were not statistically significant against the metformin comparator. Within the COVID-19 cohort, prior semaglutide exposure was associated with lower composite COVID-19 severity across the full study period, reaching significance in both vaccinated (RR 0.81, *P* = .04) and unvaccinated (RR 0.76, *P* < .001) patients, and during the pandemic period ending May 11, 2023, reaching significance in unvaccinated patients (RR 0.76, *P* = .001) but not vaccinated patients (RR 0.85, *P* = .20). When restricting to those treated with standard-of-care therapeutics during the COVID-19 pandemic era, semaglutide users had significantly lower 30-day composite risk among those receiving Paxlovid within ±7 days of infection (9.6% vs 12.7%; RR 0.76, *P* = .02). Among those initiating corticosteroids ±7 days of infection, the composite outcome remained significantly lower (27.3% vs 36.3%; RR 0.75, *P* = .007). Among the influenza cohort, semaglutide was associated with a reduced risk of the composite outcome among unvaccinated patients (RR 0.69, *P* < .001), but this trend was not statistically significant among vaccinated patients (RR 0.74, *P* = .06). Notably, semaglutide use was associated with significantly lower composite risk among Medicare-eligible adults aged ≥65 years for both COVID-19 (11.2% vs 12.9%; RR 0.86, *P* = .04) and influenza (17.0% vs 23.7%; RR 0.72, *P* = .003). Finally, composite risk did not vary significantly across pre-infection weight loss strata of <5%, 5%–15%, and ≥15% for COVID (*P* = .89) or flu (*P* = .54), or across dosage strata of 0.25–0.5, 1.0, and 1.7–2.4 mg/week for COVID (*P* = .50) or flu (*P* = .32), suggesting semaglutide-associated lower COVID and influenza severity may not be related to substantial weight loss or maximal dosing. Taken together, this study motivates prospective evaluation of low-dose semaglutide to blunt the severity of respiratory viral infections.

## Introduction

Respiratory viral infections, including coronavirus disease 2019 (COVID-19) and seasonal influenza, remain major contributors to global morbidity and mortality [1]. A consistent and reproducible observation across pandemics and endemic respiratory viruses is the disproportionate burden of severe disease among individuals with underlying metabolic dysfunction [2, 3]. Obesity and type 2 diabetes are associated with higher risks of hospitalization, respiratory failure, and death, driven by a combination of chronic low-grade inflammation, impaired innate and adaptive immune responses, endothelial dysfunction, and prothrombotic states [4, 5]. These pathophysiological features collectively amplify host susceptibility to severe viral illness and complicate recovery.

The intersection between metabolic disease and infectious outcomes has renewed interest in whether therapies targeting metabolic dysfunction may also modify the clinical course of viral infections [6]. Among these, glucagon-like peptide-1 receptor agonists (GLP-1RAs) have emerged as a cornerstone of treatment for type 2 diabetes and obesity [7]. Semaglutide, a long-acting GLP-1RA, produces substantial improvements in glycemic control, body weight, and cardiometabolic risk factors, and has demonstrated reductions in major adverse cardiovascular events in large randomized trials [8, 9]. Beyond these established effects, GLP-1 receptor signaling has been implicated in pathways relevant to infection severity, including attenuation of systemic inflammation, modulation of macrophage and T-cell activity, improvement in endothelial function, and potential effects on pulmonary and vascular biology [10].

Preclinical and translational studies suggest that GLP-1 receptor activation may dampen inflammatory cytokine signaling and improve tissue resilience under stress conditions [10]. Observational clinical data have also hinted at reduced adverse outcomes in patients treated with GLP-1RAs during acute illnesses [11–13], although findings have been inconsistent and often limited by sample size, heterogeneity in comparator groups, and lack of longitudinal follow-up [14, 15]. Importantly, it remains unclear whether any observed benefit reflects drug-specific mechanisms or broader improvements in metabolic health that are shared across multiple therapeutic classes.

Real-world evidence derived from large-scale electronic health record data provides an opportunity to evaluate these associations in diverse patient populations under routine care conditions. Such analyses can capture longitudinal outcomes across multiple clinically relevant endpoints, including mortality, hospitalization, and escalation of care, while enabling comparisons across different therapeutic contexts. In this study, we evaluated the association between semaglutide use and severity of COVID-19 and influenza in large, propensity-matched cohorts. By focusing on comparisons with patients receiving metformin but not GLP-1 receptor agonists, we aimed to assess whether semaglutide use is associated with reduced risk of severe infection in routine clinical practice.

## Materials and methods

### Study design and cohort definition

For this retrospective cohort study, two parallel cohorts were constructed: one for COVID-19 and one for influenza. Adults aged 18 years or older with a positive infection during the study period (January 1, 2020 to December 31, 2025) were eligible. COVID-19 infection was defined as a positive SARS-CoV-2 PCR or antigen test result or an ICD-10 code of U07.1. Influenza infection was defined as a positive influenza PCR or antigen test result (influenza A or B) or an ICD-10 code of J09.x, J10.x, or J11.x. For each cohort, each patient contributed a single index date corresponding to the first qualifying infection occurring after all cohort inclusion, exclusion, and exposure criteria were met.

### Inclusion and exclusion criteria

Patients were required to have at least 12 months of baseline electronic health record data prior to the index infection, defined as documentation of at least one clinical encounter occurring more than 12 months before the index infection date and at least one clinical encounter occurring within 12 months of the index infection date. Exclusion criteria, applied symmetrically to both cohorts, were: documented prior infection with the same pathogen in the 6 months prior to index (to ensure incident events); active malignancy in the 24 months prior, defined as a diagnosis of malignant neoplasms or administration of chemotherapy; organ transplant at any time prior; active immunosuppressive medication exposure in the 3 months prior; HIV or AIDS at any time prior; end-stage renal disease or dialysis dependence in the 12 months prior; and pregnancy in the 12 months prior. The relevant codes and definitions used to determine exclusion criteria are defined in Supplementary Table 1.

### Exposure definition

This study used an active-comparator design, comparing semaglutide users with metformin users to reduce confounding by indication and healthcare utilization differences [16, 17]. Active semaglutide exposure was defined as 2 or more orders or administrations of any semaglutide formulation (Ozempic, Wegovy, Rybelsus) in the 12 months prior to the index infection. The active comparator cohort comprised metformin users, defined as 2 or more orders or administrations of metformin in the 12 months prior to the index infection, with no orders or administrations of any GLP-1 receptor agonist (semaglutide, liraglutide, dulaglutide, exenatide) or tirzepatide at any time prior to the index infection. Exposure status was assigned at the index date based on the 12-month baseline window and was not redefined during follow-up; initiation of a GLP-1 receptor agonist by a metformin comparator patient after the index date, or discontinuation of semaglutide after index, did not change the baseline exposure assignment.

### Covariates and propensity score matching

Baseline covariates were assessed within the 12 months prior to the index infection date. Demographic covariates included age category (18–24, 25–34, 35–44, 45–54, 55–64, 65–74, 75–84, 85, and older), sex, race, and ethnicity. Anthropometric and metabolic covariates included BMI category (underweight, normal, overweight, class 1 obesity, class 2 obesity, class 3 obesity, unknown) and HbA1c category (normal, pre-diabetic, diabetic, unknown). Infection-context covariates included documentation of a COVID-19 or influenza vaccine in the 12 months prior to the index infection (any vaccination vs none); documentation of a prior infection with the relevant pathogen between 12 and 6 months before index (with infections within 6 months captured by the exclusion criterion above); and use of a guideline-recommended antiviral therapy within ±7 days of the index infection (nirmatrelvir/ritonavir, molnupiravir, or remdesivir for COVID-19; oseltamivir, zanamivir, peramivir, or baloxavir for influenza). For the COVID-19 cohort, an additional contextual variable was included: SARS-CoV-2 variant era at the time of index, defined by the dominant circulating variant (pre-Delta, before July 2021; Delta, July through December 2021; Omicron BA.1/BA.2, January through June 2022; Omicron BA.4/BA.5, July through December 2022; or Omicron XBB and later, January 2023 onward). Comorbidity indicators captured anxiety, asthma, atrial fibrillation, chronic kidney disease stage 3 or 4, chronic liver disease, chronic obstructive pulmonary disease, coronary artery disease, depression, heart failure, hypertension, NAFLD or MASH, obesity, history of stroke, type 2 diabetes mellitus, and current and former tobacco use. Healthcare utilization indicators captured any office or outpatient visit or hospital inpatient stay within the 12-month baseline period. The relevant codes used to determine baseline covariates are defined in Supplementary Table 1. Propensity-score adjustment was used to control confounding across the high-dimensional covariate space, an approach shown to improve confounding control in healthcare database studies [18]. Propensity scores were estimated via logistic regression including all baseline covariates listed above; the COVID-19 propensity model additionally included variant era [19, 20]. One-to-one nearest-neighbor matching without replacement was performed on the logit of the propensity score using a caliper of 0.2 standard deviations of the logit of the propensity score [20, 21]. Propensity-matched, active-comparator real-world analyses of this kind have been shown to approximate the findings of randomized trials, supporting the validity of this design for estimating treatment associations [22].

### Outcome definitions

Clinical outcomes were assessed within 30 days following the index infection date. Primary outcomes included all-cause mortality, hospitalization, emergency department visit, intensive care unit admission, and mechanical ventilation. A composite outcome was defined as the occurrence of all-cause mortality, hospitalization, intensive care unit admission, or mechanical ventilation within 30 days of index. Emergency department visits were assessed as a separate outcome and were not included in the composite. Cardiac, pulmonary, and renal complications were similarly assessed within the 30-day window using ICD-10 code groupings. Cardiac complications comprised acute coronary syndrome (myocardial infarction, angina, coronary thrombosis), sudden cardiac event (cardiac arrest, ventricular tachycardia or fibrillation, sudden cardiac death, atrioventricular block), heart failure or shock (acute heart failure, decompensation, cardiogenic shock), cerebrovascular event (ischemic stroke, hemorrhagic stroke, transient ischemic attack), and thromboembolic event (pulmonary embolism, deep vein thrombosis, systemic or peripheral arterial thromboembolism). Pulmonary complications comprised oxygen requirement, acute respiratory distress syndrome, and mechanical ventilation. Renal complications comprised acute kidney injury and dialysis or renal replacement therapy. As a negative control outcome to assess residual confounding, incident urinary tract infection within 30 days of the index infection was ascertained, selected because it has no plausible causal relationship with pre-infection semaglutide exposure while remaining subject to ascertainment and confounding similar to the primary outcomes [23]. The relevant codes used to determine outcomes are defined in Supplementary Table 1.

### Sub-analysis by corticosteroid use

A sub-analysis was performed to assess whether the association between semaglutide and clinical outcomes was modified by corticosteroid use at infection. Corticosteroid use was defined as an order or administration of any systemic corticosteroid within ±7 days of the index infection. Eligible medications included dexamethasone, methylprednisolone, prednisone, prednisolone, hydrocortisone, betamethasone, triamcinolone, cortisone, fludrocortisone, deflazacort, and oral budesonide. Inhaled and topical corticosteroid formulations were excluded.

### Stratified analyses within co-intervention exposure groups

Stratified analyses were performed within the propensity-matched COVID-19 cohort, restricted to the pandemic era (infections before May 11, 2023). For each analysis, the matched cohort was restricted to patients meeting a pre-specified co-intervention or vaccination criterion in both groups: (i) initiation of systemic corticosteroids (dexamethasone, methylprednisolone, prednisone, prednisolone, hydrocortisone, betamethasone, triamcinolone, cortisone, fludrocortisone, deflazacort, and oral budesonide) within ±7 days of COVID-19 index infection; (ii) initiation of Paxlovid (nirmatrelvir/ritonavir) within ±7 days of COVID-19 index infection; and (iii) documented COVID-19 vaccination within the 12 months prior to COVID-19 index infection.

### Within-Cohort sub-analyses: weight loss and dose

Two sub-analyses were conducted to assess whether 30-day composite outcome rates among semaglutide-treated patients vary with markers of drug response. These analyses were performed on the pre-matched semaglutide cohort and did not involve a comparator cohort. For the weight loss sub-analysis, percent weight loss was calculated as the difference between the weight measurement closest to first semaglutide order or administration (within the 90 days prior) and the weight measurement closest to the index infection date (within the 90 days prior), expressed as a percentage of the baseline weight. Patients without both weight measurements available were excluded. Weight loss strata were defined as: <5%, 5%–15%, and ≥15%. For the semaglutide dose sub-analysis, the subcutaneous semaglutide dose closest to the infection date within the 12-month baseline period was identified. The dose analysis was restricted to patients receiving subcutaneous semaglutide (Ozempic or Wegovy); patients whose closest pre-infection prescription was for oral semaglutide (Rybelsus) were excluded, since the oral formulation uses a distinct dosing scale (3, 7, or 14 mg daily) that is not directly comparable to the weekly subcutaneous milligram dose. Patients without subcutaneous dose information available were excluded. Strata were defined according to standard subcutaneous semaglutide dosing across both Ozempic and Wegovy formulations: 0.25–0.5 mg/week (titration phase), 1.0 mg/week (Wegovy mid-titration and Ozempic standard maintenance), and 1.7–2.4 mg/week (including Wegovy therapeutic maintenance and Ozempic maximum) [8, 24].

### Statistical analysis

Baseline characteristics were summarized as count with percent for categorical variables. Standardized mean differences were used to assess pre- and post-matching balance [25]. Risk ratios with 95% confidence intervals were calculated using normal approximation on the log-risk-ratio scale [26]. *P* values for between-group comparisons were derived from the Pearson chi-square test of independence without continuity correction [27]. For comparisons in which a group had zero events, a 0.5 continuity correction was applied to the risk ratio and confidence interval. For within-cohort sub-analyses by weight loss and subcutaneous semaglutide dose stratum, the event rate was summarized as a proportion with a 95% Wilson score confidence interval [28]; *P* values were derived from the Cochran-Armitage test for trend [29, 30]. Two-sided *P* values below 0.05 were considered statistically significant. To account for multiple comparisons across the outcomes, adjusted *P* values are also reported using Benjamini-Hochberg false discovery rate [31]. All analyses were performed in Python 3.14, with propensity scores estimated using a logistic regression model fit in scikit-learn 1.9.0, and risk ratios, confidence intervals, and *P*-values computed using SciPy, NumPy, and pandas. The study was planned and is reported in accordance with recognized frameworks for real-world evidence and pharmacoepidemiology, including the STaRT-RWE template, the RECORD-PE reporting statement, and joint ISPE/ISPOR guidance for reproducible healthcare-database studies [32–34].

### Data source

This study analyzed de-identified EHR data from academic medical centers in the United States via the nference nSights Analytics Platform. Prior to analysis, all data underwent expert determination de-identification satisfying HIPAA Privacy Rule requirements [45 CFR §164.514(b)(1)], employing a multi-layered transformation approach for both structured data (cryptographic hashing of identifiers, date-shifting, geographic truncation) and unstructured clinical text (ensemble deep learning and rule-based methods with >99% recall for personally identifiable information detection). nference established secure data environments within each participating center, housing these de-identified patient data governed by expert determination. These de-identified data environments were specifically designed to enable data access and analysis without requiring Institutional Review Board oversight, approval, or exemption confirmation. Accordingly, informed consent and IRB review were not required for this study.

### Data availability

This study involves the analysis of de-identified Electronic Health Record (EHR) data via the nference nSights Federated Clinical Analytics Platform (nSights). Data shown and reported in this manuscript were extracted from this environment using an established protocol for data extraction, aimed at preserving patient privacy. The data has been de-identified pursuant to an expert determination in accordance with the HIPAA Privacy Rule. Any data beyond what is reported in the manuscript, including but not limited to the raw EHR data, cannot be shared or released due to the parameters of the expert determination to maintain the data de-identification. The corresponding author should be contacted for additional details regarding nSights.

### De-identification and HIPAA compliance certification

Prior to analysis, all EHR data were de-identified under an expert determination consistent with the Health Insurance Portability and Accountability Act (HIPAA) Privacy Rule [45 CFR §164.514(b)(1)]. The de-identification methodology employed a multi-layered transformation approach to both structured and unstructured data fields [35]. In structured data, direct identifiers including patient names and precise geographic locations were excluded entirely, while indirect identifiers underwent specific transformations: patient identifiers, medical record numbers, and accession numbers were replaced with one-way cryptographic hashes using confidential salts to preserve linkage across patient encounters; all dates were shifted backward by patient-specific random offsets (1–31 days) to preserve temporal relationships while obscuring exact event timing; the ZIP codes were truncated to two-digit state-level resolution; and continuous variables including age, height, weight, and body mass index were thresholded to prevent identification of extreme values (for example, ages ≥89 years transformed to “89+” and BMI >40 transformed to “40+”). In unstructured clinical text, an ensemble de-identification system that combines attention-based deep learning models with rule-based methods achieved an estimated >99% recall for personally identifiable information (PII) detection, with detected identifiers replaced by plausible fictional surrogates [35].

### Data harmonization

To address heterogeneity in EHR data, we harmonized clinical variables including medications, anthropometric measurements, and diagnoses to standardized concepts. For medications, we first constructed a standardized drug concept database combining the nSights knowledge graph with RXNorm (https://www.nlm.nih.gov/research/umls/rxnorm/index.html) hierarchies to capture ingredient, brand, and dose-specific information [36]. EHR medication records were matched using a hierarchical approach prioritizing RXNorm codes when available, followed by ingredient-level matching, and finally natural language processing and pattern matching on free-text medication orders when structured codes were absent. For anthropometric measurements (height, weight, BMI), we created a unified vocabulary from SNOMED (https://www.snomed.org/, https://athena.ohdsi.org) and LOINC (https://loinc.org/) terminologies and matched EHR measurement descriptions using standardized text matching algorithms with abbreviation expansion and synonym resolution; ambiguous mappings were resolved using OpenAI GPT-4o (https://platform.openai.com/docs/models/gpt-4o) with summary statistics as context, followed by manual verification. For diagnoses, we developed a hierarchical disease concept database from the nSights knowledge graph and matched EHR diagnosis descriptions and codes by identifying the most specific common child concept in the hierarchy. This approach enabled consistent identification of clinical entities while preserving granularity where available.

### Code availability

The analysis code is not publicly available. The corresponding author should be contacted for additional details.

## Results

### Cohort eligibility criteria and propensity matching

**Figure 1:**
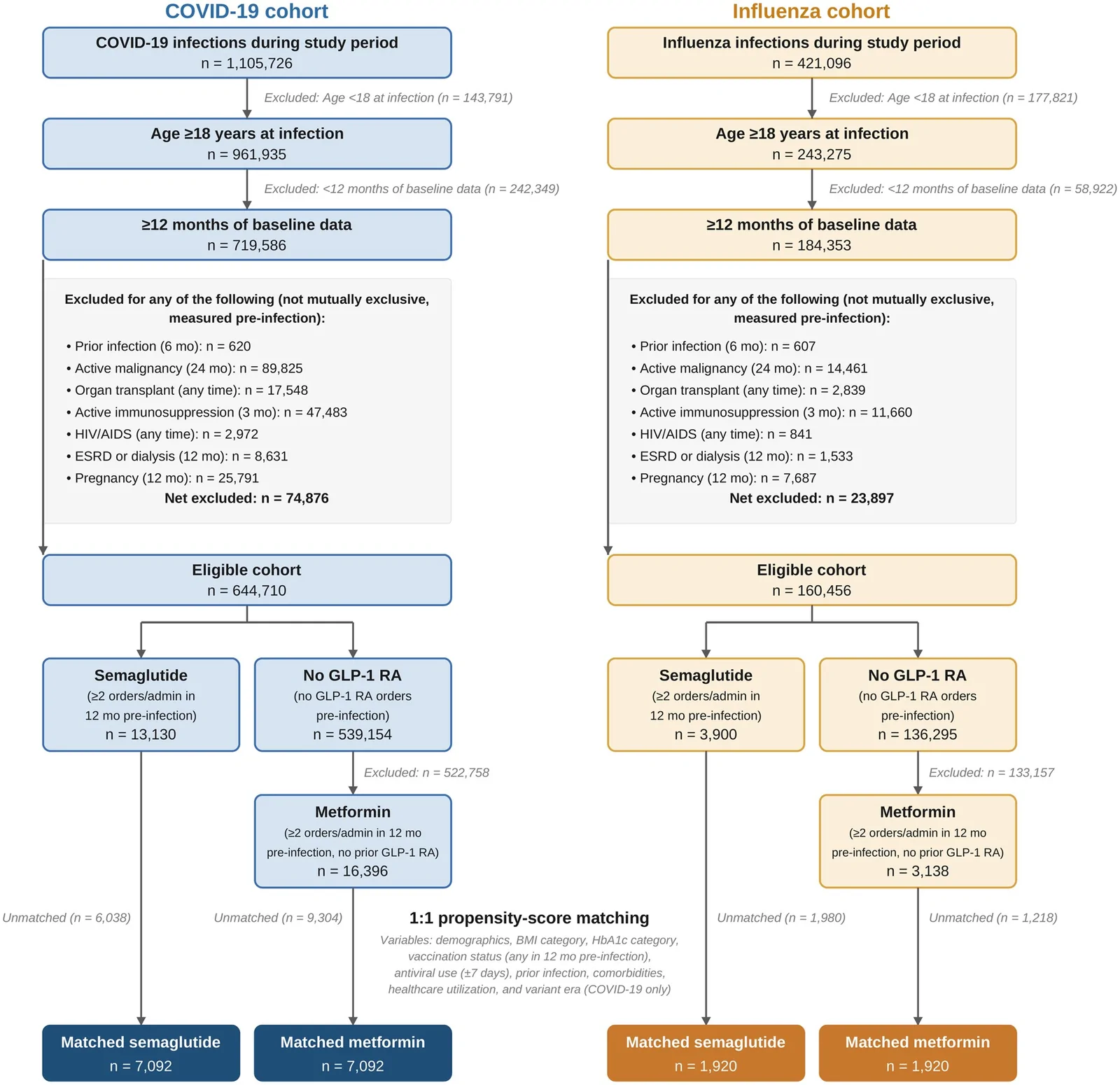
Cohort derivation for COVID-19 and influenza analyses. Flow diagram illustrating cohort selection for the COVID-19 (left) and influenza (right) analyses during the study period (January 1, 2020 to December 31, 2025). Patients with documented infection were restricted to adults (≥18 years) with at least 12 months of baseline data. Individuals were excluded for selected pre-existing conditions or exposures, including recent prior infection, active malignancy, organ transplant, immunosuppression, HIV/AIDS, end-stage renal disease, or pregnancy. From the eligible cohorts, patients with evidence of semaglutide exposure (≥2 orders or administrations within 12 months prior to infection) were identified and compared with a metformin comparator group, defined as patients with ≥2 metformin orders or administrations within 12 months prior to infection and no prior GLP-1 receptor agonist exposure. Propensity score matching (1:1) was performed, yielding matched cohorts of 7092 semaglutide-treated and 7092 metformin-treated patients for COVID-19, and 1920 semaglutide-treated and 1920 metformin-treated patients for influenza. Counts for exclusion criteria are not mutually exclusive.

Cohort derivation is summarized in Fig. 1. Across the study period (January 1, 2020 to December 31, 2025), 644 710 adults with a documented COVID-19 infection and 160 456 adults with a documented influenza infection met initial eligibility criteria. Among the eligible COVID-19-infected population, 13 130 patients had ≥2 orders or administrations of semaglutide in the 12 months before the index infection (“exposed”), and 16 396 patients met the metformin active-comparator definition (“comparator”). Among the eligible influenza-infected population, 3900 patients had exposure to semaglutide and 3138 patients met the metformin active-comparator definition.

After 1:1 propensity matching on demographic and clinical covariates, the COVID-19 cohorts each comprised 7092 matched patients and the influenza cohorts each comprised 1920 matched patients. The largest imbalances before matching were observed for variant era, age group, obesity, and BMI category, with standardized mean differences (SMDs) up to approximately 0.75 and no covariate exceeding 1.0 (Table 1). After matching, all matching variables in both cohorts achieved SMDs below 0.10, indicating baseline balance (Table 2; Supplementary Fig. 1). In the COVID-19 cohort, the proportion of patients aged ≥65 years was 40.2% (semaglutide) vs 39.3% (comparator, SMD 0.035), the proportion with type 2 diabetes was 72.8% (semaglutide) vs 73.5% (comparator, SMD 0.016), and the proportion with obesity was 41.9% (semaglutide) vs 41.7% (comparator, SMD 0.004). Similar balance was achieved in the influenza cohort, where the proportion aged 65 years or older was 34.1% (semaglutide) vs 33.6% (comparator, SMD 0.058), the proportion with type 2 diabetes was 70.6% (semaglutide) vs 69.2% (comparator, SMD 0.031), and the proportion with obesity was 37.4% (semaglutide) vs 37.3% (comparator, SMD 0.001). Time since first exposure, reported in Tables 1 and 2 for description only, was not used as a matching covariate and remained imbalanced after matching because metformin and semaglutide entered clinical use in different eras.

**Table 1:** Pre-matching baseline characteristics of the semaglutide exposure and metformin comparator cohorts, COVID-19 and influenza.

Characteristic	COVID-19 cohort			Influenza cohort		
	Semaglutide (*n* = 13 130)	Metformin (*n* = 16 396)	SMD	Semaglutide (*n* = 3900)	Metformin (*n* = 3138)	SMD
**Demographics**						
** Age group, *n* (%)**			0.716			0.674
** 18–24**	139 (1.1)	267 (1.6)		87 (2.2)	97 (3.1)	
** 25–34**	749 (5.7)	671 (4.1)		345 (8.8)	196 (6.2)	
** 35–44**	1927 (14.7)	1103 (6.7)		751 (19.3)	287 (9.1)	
** 45–54**	3075 (23.4)	1851 (11.3)		973 (24.9)	407 (13.0)	
** 55–64**	3624 (27.6)	3240 (19.8)		899 (23.1)	656 (20.9)	
** 65–74**	2657 (20.2)	4679 (28.5)		633 (16.2)	806 (25.7)	
** 75–84**	886 (6.7)	3736 (22.8)		192 (4.9)	553 (17.6)	
** 85+**	73 (0.6)	849 (5.2)		20 (0.5)	136 (4.3)	
** Sex, *n* (%)**			0.377			0.382
** Female**	8916 (67.9)	8145 (49.7)		2709 (69.5)	1603 (51.1)	
** Male**	4212 (32.1)	8250 (50.3)		1191 (30.5)	1535 (48.9)	
** Unknown**	<11	<11		0 (0.0)	0 (0.0)	
** Race, *n* (%)**			0.227			0.253
** White**	10 168 (77.4)	13 041 (79.5)		2980 (76.4)	2372 (75.6)	
** Black or African American**	2049 (15.6)	1587 (9.7)		598 (15.3)	319 (10.2)	
** Asian**	231 (1.8)	592 (3.6)		82 (2.1)	150 (4.8)	
** Native American or Pacific Islander**	127 (1.0)	158 (1.0)		42 (1.1)	42 (1.3)	
** Other/Mixed**	210 (1.6)	492 (3.0)		73 (1.9)	136 (4.3)	
** Unknown**	345 (2.6)	526 (3.2)		125 (3.2)	119 (3.8)	
** Ethnicity, *n* (%)**			0.177			0.176
** Hispanic or Latino**	601 (4.6)	1055 (6.4)		216 (5.5)	251 (8.0)	
** Not Hispanic or Latino**	10 794 (82.2)	13 999 (85.4)		3087 (79.2)	2568 (81.8)	
** Unknown**	1735 (13.2)	1342 (8.2)		597 (15.3)	319 (10.2)	
**Anthropometrics and metabolic status**						
** BMI category, *n* (%)**			0.549			0.513
** Underweight (<18.5)**	13 (0.1)	80 (0.5)		<11	15 (0.5)	
** Normal (18.5–24.9)**	557 (4.2)	2174 (13.3)		182 (4.7)	426 (13.6)	
** Overweight (25–29.9)**	2261 (17.2)	4801 (29.3)		706 (18.1)	924 (29.4)	
** Class 1 obesity (30–34.9)**	3411 (26.0)	4325 (26.4)		1015 (26.0)	795 (25.3)	
** Class 2 obesity (35–39.9)**	3199 (24.4)	2449 (14.9)		894 (22.9)	485 (15.5)	
** Class 3 obesity (≥40)**	1648 (12.6)	971 (5.9)		503 (12.9)	221 (7.0)	
** Unknown**	2041 (15.5)	1596 (9.7)		597 (15.3)	272 (8.7)	
** HbA1c category, *n* (%)**			0.331			0.406
** Normal (4.0–5.6)**	2226 (17.0)	1533 (9.3)		719 (18.4)	277 (8.8)	
** Pre-diabetic (5.7–6.4)**	2473 (18.8)	4341 (26.5)		639 (16.4)	771 (24.6)	
** Diabetic (6.5–16.0)**	4640 (35.3)	7018 (42.8)		1217 (31.2)	1307 (41.7)	
** Unknown**	3791 (28.9)	3504 (21.4)		1325 (34.0)	783 (25.0)	
**Infection context**						
** Vaccinated, *n* (%)**	2970 (22.6)	3824 (23.3)	0.017	840 (21.5)	715 (22.8)	0.030
** Variant era, *n* (%)**			0.750			–
** Pre-Delta**	583 (4.4)	3125 (19.1)		–	–	
** Delta**	1007 (7.7)	2594 (15.8)		–	–	
** Omicron BA.1/BA.2**	1178 (9.0)	2517 (15.4)		–	–	
** Omicron BA.4/BA.5**	1577 (12.0)	2555 (15.6)		–	–	
** Omicron XBB or later**	8785 (66.9)	5605 (34.2)		–	–	
** Antiviral ±7 days of infection, *n* (%)**	6814 (51.9)	6002 (36.6)	0.312	2479 (63.6)	1959 (62.4)	0.024
** Prior infection, *n* (%)**	515 (3.9)	431 (2.6)	0.073	51 (1.3)	35 (1.1)	0.018
**Comorbidities, *n* (%)**						
** Anxiety**	3728 (28.4)	3103 (18.9)	0.224	1192 (30.6)	626 (19.9)	0.246
** Asthma**	1759 (13.4)	1395 (8.5)	0.157	552 (14.2)	283 (9.0)	0.161
** Atrial fibrillation**	873 (6.6)	1833 (11.2)	0.160	220 (5.6)	339 (10.8)	0.189
** CKD, stage 3–4**	1437 (10.9)	2353 (14.4)	0.103	346 (8.9)	368 (11.7)	0.094
** Chronic liver disease**	1200 (9.1)	993 (6.1)	0.117	334 (8.6)	209 (6.7)	0.072
** COPD**	893 (6.8)	1532 (9.3)	0.093	261 (6.7)	342 (10.9)	0.149
** Coronary artery disease**	1843 (14.0)	3289 (20.1)	0.161	520 (13.3)	579 (18.5)	0.140
** Depression**	3538 (26.9)	3138 (19.1)	0.186	1036 (26.6)	616 (19.6)	0.165
** Heart failure**	1025 (7.8)	1781 (10.9)	0.105	299 (7.7)	350 (11.2)	0.120
** Hypertension**	8577 (65.3)	11 461 (69.9)	0.098	2303 (59.1)	2085 (66.4)	0.153
** NAFLD/MASH**	1056 (8.0)	719 (4.4)	0.152	286 (7.3)	142 (4.5)	0.119
** Obesity**	7508 (57.2)	4387 (26.8)	0.648	2223 (57.0)	810 (25.8)	0.668
** Stroke, history of**	163 (1.2)	460 (2.8)	0.111	44 (1.1)	87 (2.8)	0.119
** Tobacco use, current**	784 (6.0)	1282 (7.8)	0.073	278 (7.1)	338 (10.8)	0.128
** Tobacco use, former**	852 (6.5)	1350 (8.2)	0.067	255 (6.5)	255 (8.1)	0.061
** Type 2 diabetes**	8461 (64.4)	13 114 (80.0)	0.352	2221 (56.9)	2369 (75.5)	0.400
**Healthcare utilization (12 months pre-index), *n* (%)**						
** Office or outpatient visit**	4959 (37.8)	6848 (41.8)	0.082	1518 (38.9)	1224 (39.0)	0.002
** Hospital inpatient stay**	1119 (8.5)	2381 (14.5)	0.189	326 (8.4)	470 (15.0)	0.207
** Time (months) since first exposure, mean (SD)^a^**	16.1 (13.2)	66.1 (55.5)	−1.239	19.3 (15.1)	62.3 (54.8)	−1.070

Demographic, clinical, and healthcare utilization characteristics measured in the 12 months prior to infection, before propensity-score matching. Standardized mean differences (SMDs) compare the semaglutide and metformin cohorts within each infection cohort.

a Time (months) since first exposure is the time from the first recorded order or administration of the index medication to the index infection date, shown for description only. It was not a matching covariate.

**Table 2:** Post-matching baseline characteristics (1:1 propensity-score matched) of the semaglutide exposure and metformin comparator cohorts, COVID-19 and influenza.

Characteristic	COVID-19 cohort			Influenza cohort		
	Semaglutide (*n* = 7092)	Metformin (*n* = 7092)	SMD	Semaglutide (*n* = 1920)	Metformin (*n* = 1920)	SMD
**Demographics**						
** Age group, *n* (%)**			0.035			0.058
** 18–24**	95 (1.3)	107 (1.5)		52 (2.7)	63 (3.3)	
** 25–34**	424 (6.0)	386 (5.4)		166 (8.6)	162 (8.4)	
** 35–44**	710 (10.0)	727 (10.3)		237 (12.3)	241 (12.6)	
** 45–54**	1179 (16.6)	1196 (16.9)		335 (17.4)	336 (17.5)	
** 55–64**	1832 (25.8)	1888 (26.6)		476 (24.8)	472 (24.6)	
** 65–74**	1949 (27.5)	1906 (26.9)		463 (24.1)	440 (22.9)	
** 75–84**	830 (11.7)	808 (11.4)		172 (9.0)	179 (9.3)	
** 85+**	73 (1.0)	74 (1.0)		19 (1.0)	27 (1.4)	
** Sex, *n* (%)**			0.014			0.012
** Female**	4197 (59.2)	4147 (58.5)		1145 (59.6)	1134 (59.1)	
** Male**	2894 (40.8)	2944 (41.5)		775 (40.4)	786 (40.9)	
** Unknown**	<11	<11		0 (0.0)	0 (0.0)	
** Race, *n* (%)**			0.016			0.045
** White**	5627 (79.3)	5643 (79.6)		1487 (77.4)	1487 (77.4)	
** Black or African American**	862 (12.2)	843 (11.9)		210 (10.9)	218 (11.4)	
** Asian**	178 (2.5)	189 (2.7)		61 (3.2)	66 (3.4)	
** Native American or Pacific Islander**	62 (0.9)	64 (0.9)		26 (1.4)	27 (1.4)	
** Other/Mixed**	151 (2.1)	152 (2.1)		57 (3.0)	58 (3.0)	
** Unknown**	212 (3.0)	201 (2.8)		79 (4.1)	64 (3.3)	
** Ethnicity, *n* (%)**			0.007			0.023
** Hispanic or Latino**	376 (5.3)	380 (5.4)		125 (6.5)	134 (7.0)	
** Not Hispanic or Latino**	5964 (84.1)	5975 (84.2)		1581 (82.3)	1581 (82.3)	
** Unknown**	752 (10.6)	737 (10.4)		214 (11.1)	205 (10.7)	
**Anthropometrics and metabolic status**						
** BMI category, *n* (%)**			0.023			0.059
** Underweight (<18.5)**	<11	13 (0.2)		<11	<11	
** Normal (18.5–24.9)**	458 (6.5)	453 (6.4)		145 (7.6)	135 (7.0)	
** Overweight (25–29.9)**	1642 (23.2)	1621 (22.9)		463 (24.1)	471 (24.5)	
** Class 1 obesity (30–34.9)**	1968 (27.7)	2019 (28.5)		545 (28.4)	526 (27.4)	
** Class 2 obesity (35–39.9)**	1516 (21.4)	1478 (20.8)		360 (18.8)	384 (20.0)	
** Class 3 obesity (≥40)**	726 (10.2)	718 (10.1)		207 (10.8)	203 (10.6)	
** Unknown**	772 (10.9)	790 (11.1)		199 (10.4)	197 (10.3)	
** HbA1c category, *n* (%)**			0.012			0.027
** Normal (4.0–5.6)**	907 (12.8)	880 (12.4)		212 (11.0)	227 (11.8)	
** Pre-diabetic (5.7–6.4)**	1528 (21.5)	1537 (21.7)		406 (21.1)	397 (20.7)	
** Diabetic (6.5–16.0)**	2916 (41.1)	2936 (41.4)		760 (39.6)	750 (39.1)	
** Unknown**	1741 (24.5)	1739 (24.5)		542 (28.2)	546 (28.4)	
**Infection context**						
** Vaccinated, *n* (%)**	1646 (23.2)	1687 (23.8)	0.014	407 (21.2)	399 (20.8)	0.010
** Variant era, *n* (%)**			0.047			–
** Pre-Delta**	547 (7.7)	576 (8.1)		–	–	
** Delta**	746 (10.5)	832 (11.7)		–	–	
** Omicron BA.1/BA.2**	857 (12.1)	860 (12.1)		–	–	
** Omicron BA.4/BA.5**	1059 (14.9)	1070 (15.1)		–	–	
** Omicron XBB or later**	3883 (54.8)	3754 (52.9)		–	–	
** Antiviral ±7 days of infection, *n* (%)**	3329 (46.9)	3186 (44.9)	0.040	1202 (62.6)	1192 (62.1)	0.011
** Prior infection, *n* (%)**	219 (3.1)	219 (3.1)	0.000	22 (1.1)	17 (0.9)	0.026
**Comorbidities, *n* (%)**						
** Anxiety**	1674 (23.6)	1643 (23.2)	0.010	478 (24.9)	472 (24.6)	0.007
** Asthma**	808 (11.4)	768 (10.8)	0.018	215 (11.2)	211 (11.0)	0.007
** Atrial fibrillation**	575 (8.1)	601 (8.5)	0.013	156 (8.1)	140 (7.3)	0.031
** CKD, stage 3–4**	921 (13.0)	943 (13.3)	0.009	205 (10.7)	203 (10.6)	0.003
** Chronic liver disease**	527 (7.4)	530 (7.5)	0.002	140 (7.3)	145 (7.6)	0.010
** COPD**	554 (7.8)	559 (7.9)	0.003	167 (8.7)	164 (8.5)	0.006
** Coronary artery disease**	1205 (17.0)	1181 (16.7)	0.009	310 (16.1)	303 (15.8)	0.010
** Depression**	1663 (23.4)	1601 (22.6)	0.021	459 (23.9)	445 (23.2)	0.017
** Heart failure**	630 (8.9)	614 (8.7)	0.008	174 (9.1)	177 (9.2)	0.005
** Hypertension**	4824 (68.0)	4834 (68.2)	0.003	1221 (63.6)	1204 (62.7)	0.018
** NAFLD/MASH**	432 (6.1)	433 (6.1)	0.001	113 (5.9)	106 (5.5)	0.016
** Obesity**	2973 (41.9)	2960 (41.7)	0.004	718 (37.4)	717 (37.3)	0.001
** Stroke, history of**	116 (1.6)	111 (1.6)	0.006	35 (1.8)	34 (1.8)	0.004
** Tobacco use, current**	517 (7.3)	514 (7.2)	0.002	165 (8.6)	163 (8.5)	0.004
** Tobacco use, former**	510 (7.2)	520 (7.3)	0.005	137 (7.1)	131 (6.8)	0.012
** Type 2 diabetes**	5162 (72.8)	5211 (73.5)	0.016	1356 (70.6)	1329 (69.2)	0.031
**Healthcare utilization (12 months pre-index), *n* (%)**						
** Office or outpatient visit**	2641 (37.2)	2647 (37.3)	0.002	686 (35.7)	682 (35.5)	0.004
** Hospital inpatient stay**	753 (10.6)	764 (10.8)	0.005	211 (11.0)	210 (10.9)	0.002
** Time (months) since first exposure, mean (SD)^a^**	16.8 (14.1)	62.9 (54.6)	−1.156	20.2 (16.0)	59.3 (54.4)	−0.974

Demographic, clinical, and healthcare utilization characteristics measured in the 12 months prior to infection, after 1:1 propensity-score matching. Standardized mean differences (SMDs) compare the semaglutide and metformin cohorts within each infection cohort.

a Time (months) since first exposure is the time from the first recorded order or administration of the index medication to the index infection date, shown for description only. It was not a matching covariate.

### Semaglutide use is associated with reduced 30-day clinical outcomes following COVID-19 infection

**Figure 2:**
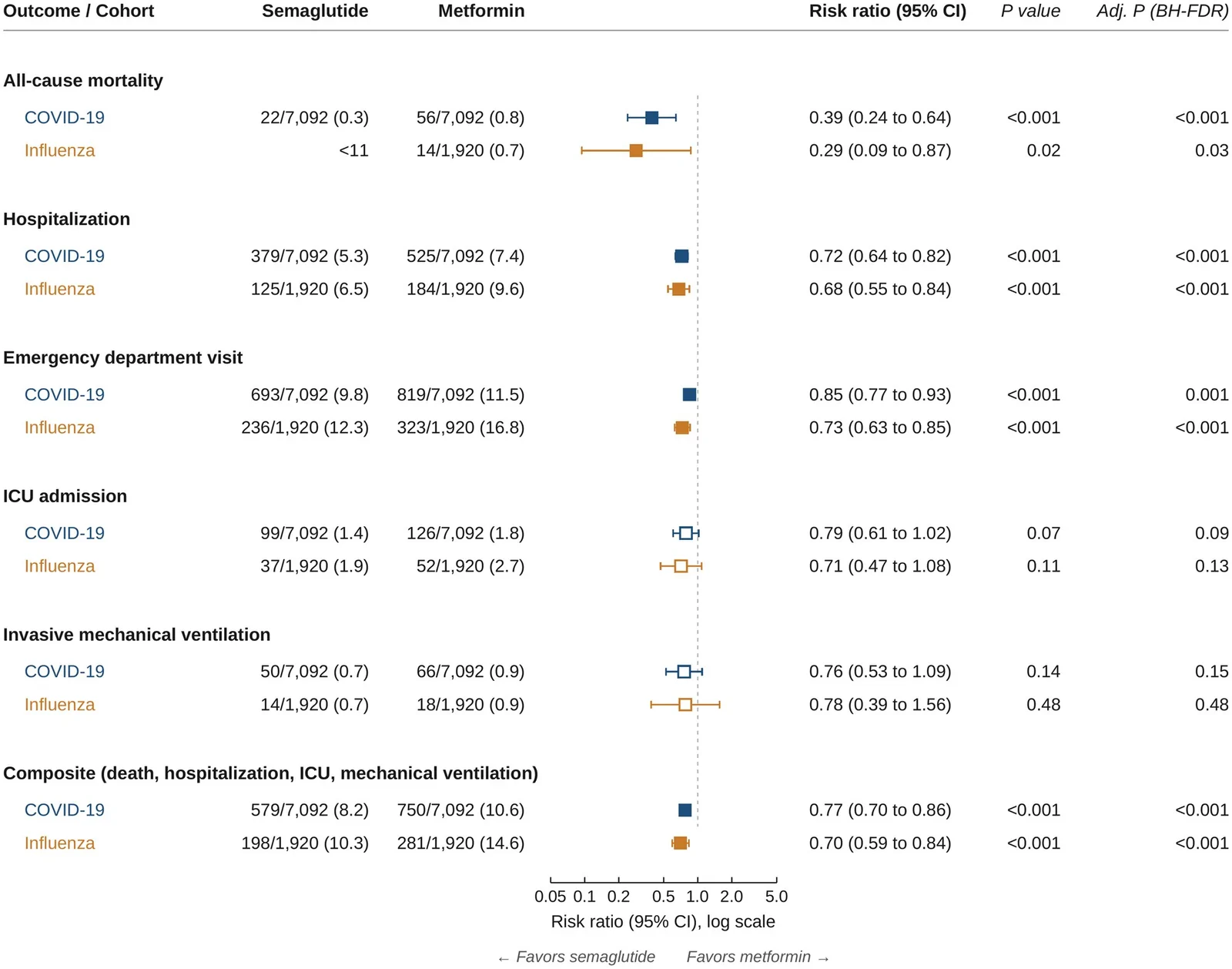
Risk of clinical outcomes following COVID-19 and influenza infection in semaglutide-treated patients vs metformin comparators. Forest plot showing risk ratios (RRs) with 95% confidence intervals for clinical outcomes within 30 days after infection in propensity-matched COVID-19 and influenza cohorts. Outcomes include all-cause mortality, hospitalization, emergency department visits, intensive care unit (ICU) admission, mechanical ventilation, and a composite outcome (death, hospitalization, ICU admission, or mechanical ventilation). Squares indicate point estimates and horizontal lines represent 95% confidence intervals; the vertical dashed line denotes a risk ratio of 1.0. Values <1.0 favor semaglutide. *P* values are derived from the Pearson chi-square test of independence. Benjamini-Hochberg adjusted *P* values are reported alongside raw values; filled squares indicate adjusted *P* < .05.

In the propensity-matched COVID-19 cohort, semaglutide use was associated with lower 30-day risk across primary severity outcomes (Fig. 2). All-cause mortality occurred in 0.3% of semaglutide-treated patients compared with 0.8% of comparators (RR 0.39, 95% CI 0.24–0.64; *P* < .001). Hospitalization occurred in 5.3% vs 7.4% (RR 0.72, 95% CI 0.64–0.82; *P* < .001), and emergency department visits occurred in 9.8% vs 11.5% (RR 0.85, 95% CI 0.77–0.93; *P* < .001). Intensive care admission occurred in 1.4% vs 1.8% (RR 0.79, 95% CI 0.61–1.02) and mechanical ventilation in 0.7% vs 0.9% (RR 0.76, 95% CI 0.53–1.09); neither reached statistical significance. The composite outcome (death, hospitalization, intensive care admission, or mechanical ventilation) occurred in 8.2% of semaglutide-treated patients compared with 10.6% of comparators (RR 0.77, 95% CI 0.70–0.86; *P* < .001). Together, these results demonstrate a coherent pattern of reduced 30-day disease severity among semaglutide-treated patients.

### Semaglutide use is associated with reduced 30-day clinical outcomes following influenza infection

In the propensity-matched influenza cohort, semaglutide use was associated with consistent reductions in 30-day risk across multiple outcomes (Fig. 2). Hospitalization occurred in 6.5% of semaglutide-treated patients compared with 9.6% of comparators (RR 0.68, 95% CI 0.55–0.84; *P* < .001). Emergency department visits occurred in 12.3% vs 16.8% (RR 0.73, 95% CI 0.63–0.85; *P* < .001). All-cause mortality was lower among semaglutide-treated patients (0.2% vs 0.7%; RR 0.29, 95% CI 0.09–0.87; *P* = .03). Intensive care admission occurred in 1.9% vs 2.7% (RR 0.71, 95% CI 0.47–1.08) and mechanical ventilation in 0.7% vs 0.9% (RR 0.78, 95% CI 0.39–1.56); neither reached statistical significance, reflecting low absolute event counts in both groups. The composite outcome occurred in 10.3% of semaglutide-treated patients compared with 14.6% of comparators (RR 0.70, 95% CI 0.59–0.84; *P* < .001).

### Semaglutide-associated risk reduction is consistent across vaccination status

**Figure 3:**
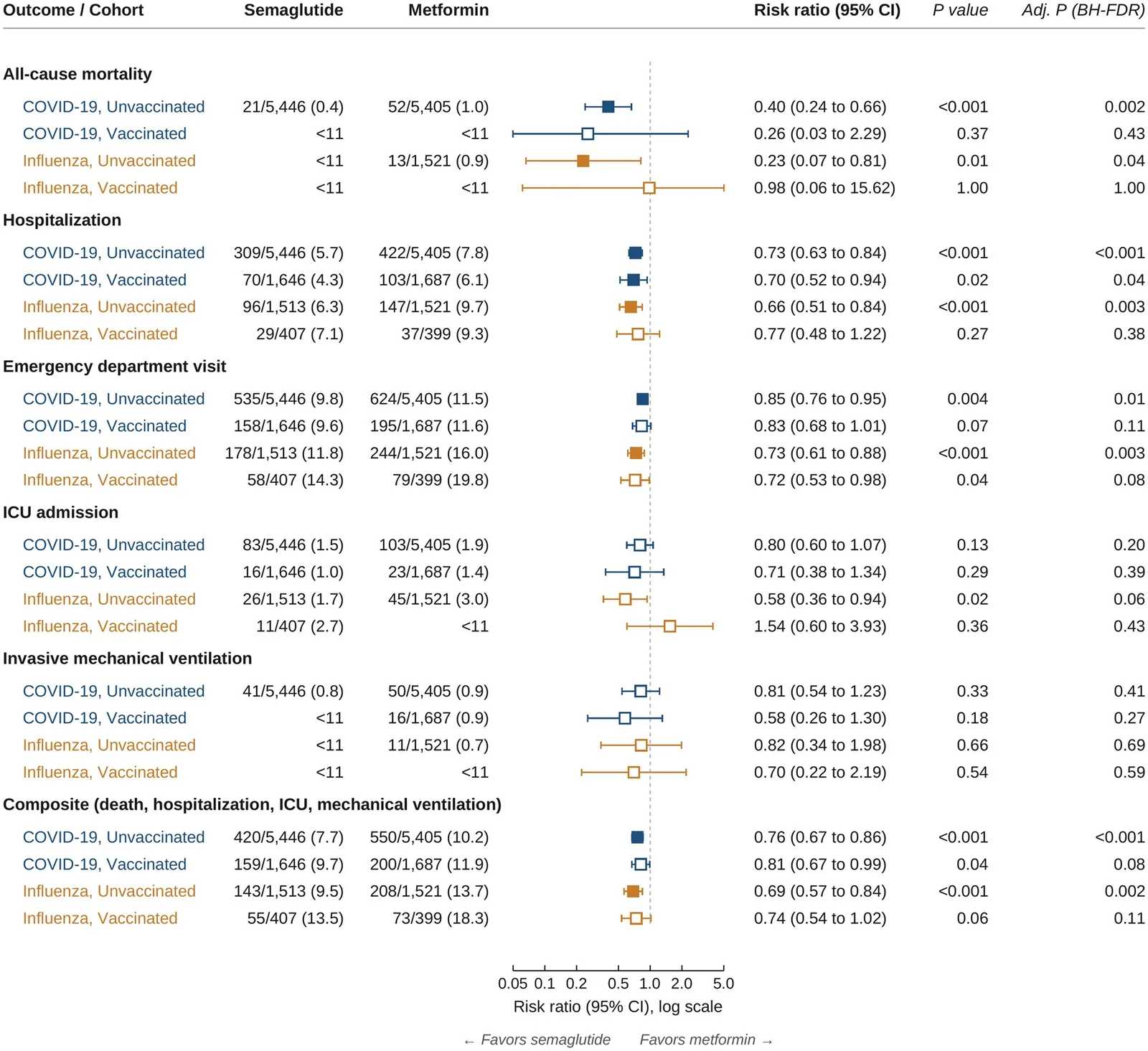
Risk of clinical outcomes following COVID-19 and influenza infection in semaglutide-treated patients vs metformin comparators, stratified by prior vaccination status. Forest plot showing risk ratios (RRs) with 95% confidence intervals for clinical outcomes within 30 days after infection in propensity-matched COVID-19 and influenza cohorts, stratified by documentation of a COVID-19 or influenza vaccine in the 12 months prior to infection. Outcomes include all-cause mortality, hospitalization, emergency department visits, intensive care unit (ICU) admission, mechanical ventilation, and a composite outcome (death, hospitalization, ICU admission, or mechanical ventilation). Squares indicate point estimates and horizontal lines represent 95% confidence intervals; the vertical dashed line denotes a risk ratio of 1.0. Values <1.0 favor semaglutide. *P* values are derived from the Pearson chi-square test of independence. Benjamini-Hochberg adjusted *P* values are reported alongside raw values; filled squares indicate adjusted *P* < .05.

To evaluate whether the observed associations were modified by prior immunization, the matched cohorts were stratified by documentation of a COVID-19 or influenza vaccine in the 12 months prior to infection (Fig. 3). Among COVID-19 patients, semaglutide use was associated with reduced composite outcome risk in both unvaccinated (*n* = 5446 vs 5405; RR 0.76, 95% CI 0.67–0.86; *P* < .001) and vaccinated (*n* = 1646 vs 1687; RR 0.81, 95% CI 0.67–0.99; *P* = .04) patients. A similar pattern was observed in the influenza cohort, with the composite outcome reduced in unvaccinated patients (*n* = 1513 vs 1521; RR 0.69, 95% CI 0.57–0.84; *P* < .001) and directionally lower but not significant in vaccinated patients (*n* = 407 vs 399; RR 0.74, 95% CI 0.54–1.02; *P* = .07). The association was similar in vaccinated and unvaccinated patients, indicating that it is not explained by higher vaccination rates.

### Semaglutide is associated with reduced 30-day acute kidney injury after viral infection

**Figure 4:**
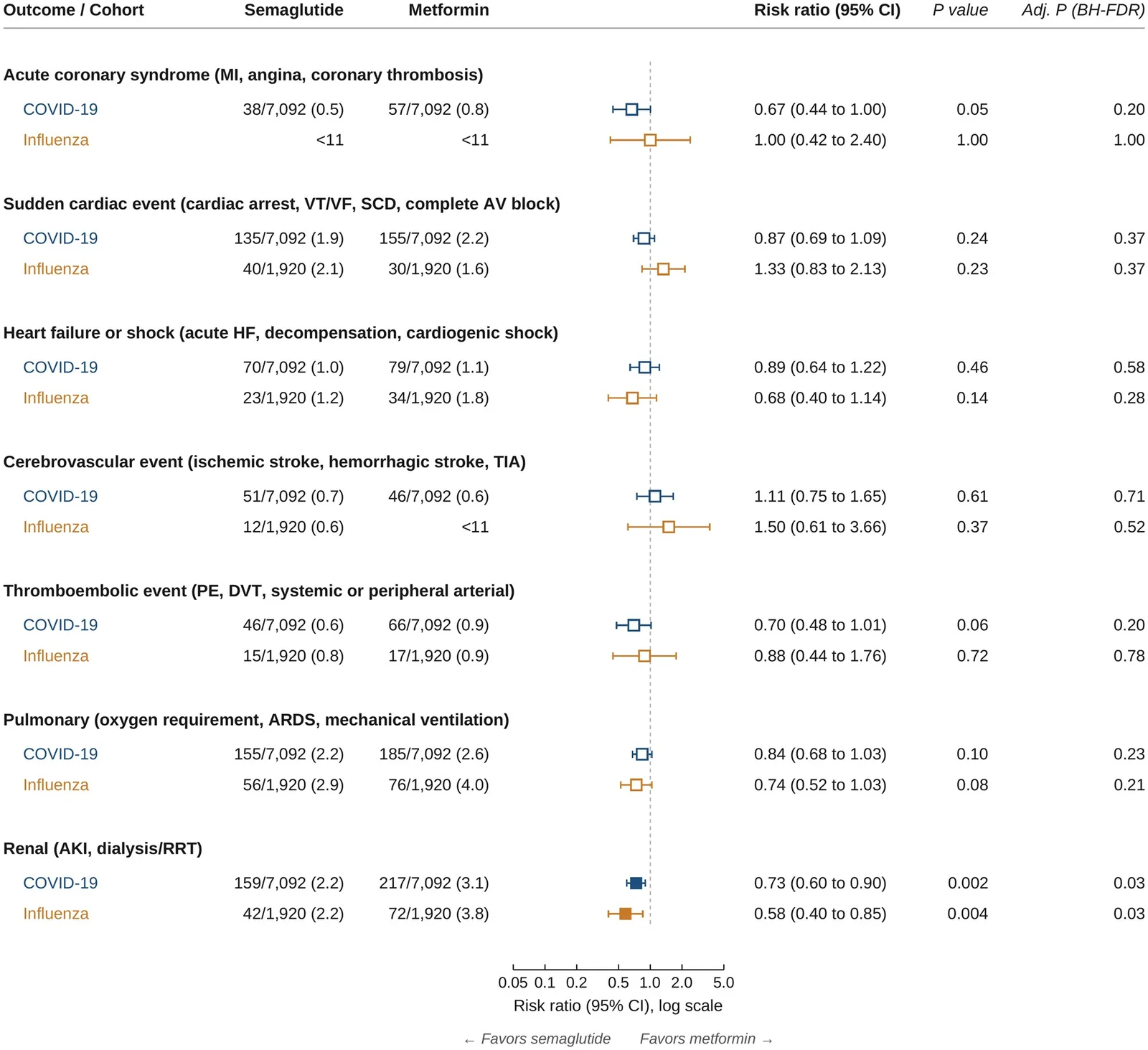
Risk of cardiac, pulmonary, and renal outcomes following COVID-19 and influenza infection in semaglutide-treated patients vs metformin comparators. Forest plot showing risk ratios (RRs) with 95% confidence intervals for cardiac, pulmonary, and renal outcomes within 30 days after infection in propensity-matched COVID-19 and influenza cohorts. Outcomes include acute coronary syndrome (myocardial infarction, angina, coronary thrombosis), sudden cardiac event (cardiac arrest, ventricular tachycardia or fibrillation, sudden cardiac death, atrioventricular block), heart failure or shock (acute heart failure, decompensation, cardiogenic shock), cerebrovascular event (ischemic stroke, hemorrhagic stroke, transient ischemic attack), thromboembolic event (pulmonary embolism, deep vein thrombosis, systemic or peripheral arterial thromboembolism), pulmonary outcomes (oxygen requirement, acute respiratory distress syndrome, mechanical ventilation), and renal outcomes (acute kidney injury, dialysis or renal replacement therapy). Squares indicate point estimates and horizontal lines represent 95% confidence intervals; the vertical dashed line denotes a risk ratio of 1.0. Values <1.0 favor semaglutide. *P* values are derived from the Pearson chi-square test of independence. Benjamini-Hochberg adjusted *P* values are reported alongside raw values; filled squares indicate adjusted *P* < .05.

Beyond the primary severity endpoints, 30-day risk of cardiac, pulmonary, and renal complications was also assessed (Fig. 4). After correction for multiple comparisons, acute kidney injury was the only organ-specific outcome that remained significantly lower in both cohorts (RR 0.73, 95% CI 0.60–0.90 in COVID-19; RR 0.58, 95% CI 0.40–0.85 in influenza). Reductions in acute coronary syndrome (RR 0.67, 95% CI 0.44–1.00 in COVID-19; RR 1.00, 95% CI 0.42–2.40 in influenza) and pulmonary outcomes including oxygen requirement, acute respiratory distress syndrome, and mechanical ventilation (RR 0.84, 95% CI 0.68–1.03 in COVID-19; RR 0.74, 95% CI 0.52–1.03 in influenza) were directionally consistent in COVID-19 but did not reach statistical significance against the metformin comparator. Estimates for sudden cardiac events, heart failure or shock, cerebrovascular events, and thromboembolic events were not statistically significant in either cohort.

### Semaglutide-associated risk reduction is consistent across age groups

To evaluate whether the observed associations were modified by age at the time of infection, the matched cohorts were stratified into three groups: 18–47, 48–64, and 65 years or older (Table 3). Among COVID-19 patients, semaglutide use was associated with a lower 30-day composite outcome risk in the 48–64 stratum (RR 0.63, 95% CI 0.52–0.75; *P* < .001) and the 65 years or older stratum (RR 0.86, 95% CI 0.75–0.99; *P* = .03); the association in the 18–47 stratum was directionally consistent but not significant (RR 0.85, 95% CI 0.64–1.14). The absolute risk reduction was largest in patients 65 years or older, where baseline event rates were highest (12.9% in comparators). All-cause mortality was significantly lower in patients 48–64 (RR 0.17, 95% CI 0.06–0.43) and not significant in patients 65 years or older (RR 0.76, 95% CI 0.40–1.42). In the influenza cohort, the composite outcome was lower in the 18–47 stratum (RR 0.43, 95% CI 0.26–0.73; *P* = .002) and the 65 years or older stratum (RR 0.72, 95% CI 0.58–0.89; *P* = .003), and not significant in the 48–64 stratum (RR 0.81, 95% CI 0.60–1.10). These findings indicate that the association is preserved among older adults at highest baseline risk of severe outcomes.

**Table 3:** Thirty-day risk of clinical outcomes following COVID-19 and influenza infection in semaglutide-treated patients versus metformin comparators, stratified by age group at infection.

Outcome	Semaglutide *n*/*N* (%)	Metformin *n*/*N* (%)	RR (95% CI)	*P*	Adj. *P* (BH-FDR)
**COVID-19 cohort—age 18–47**					
** All-cause mortality**	<11	<11	0.14 (0.01–2.73)	.12	.25
** Hospitalization**	35/1520 (2.3%)	57/1502 (3.8%)	0.61 (0.40–0.92)	.02	.06
** Emergency department visit**	110/1520 (7.2%)	118/1502 (7.9%)	0.92 (0.72–1.18)	.52	.58
** ICU admission**	<11	15/1502 (1.0%)	0.53 (0.22–1.24)	.14	.26
** Mechanical ventilation**	<11	<11	0.20 (0.02–1.69)	.12	.25
** Composite outcome**	81/1520 (5.3%)	94/1502 (6.3%)	0.85 (0.64–1.14)	.27	.39
**COVID-19 cohort—age 48–64**					
** All-cause mortality**	<11	31/2802 (1.1%)	**0.17 (0.06–0.43)**	<.001	<.001
** Hospitalization**	104/2720 (3.8%)	195/2802 (7.0%)	**0.55 (0.44–0.69)**	<.001	<.001
** Emergency department visit**	220/2720 (8.1%)	319/2802 (11.4%)	**0.71 (0.60–0.84)**	<.001	<.001
** ICU admission**	29/2720 (1.1%)	49/2802 (1.7%)	0.61 (0.39–0.96)	.03	.09
** Mechanical ventilation**	15/2720 (0.6%)	30/2802 (1.1%)	0.52 (0.28–0.96)	.03	.09
** Composite outcome**	180/2720 (6.6%)	296/2802 (10.6%)	**0.63 (0.52–0.75)**	<.001	<.001
**COVID-19 cohort—age 65+**					
** All-cause mortality**	17/2852 (0.6%)	22/2788 (0.8%)	0.76 (0.40–1.42)	.38	.49
** Hospitalization**	240/2852 (8.4%)	273/2788 (9.8%)	0.86 (0.73–1.01)	.07	.16
** Emergency department visit**	363/2852 (12.7%)	382/2788 (13.7%)	0.93 (0.81–1.06)	.28	.39
** ICU admission**	62/2852 (2.2%)	62/2788 (2.2%)	0.98 (0.69–1.38)	.90	.92
** Mechanical ventilation**	34/2852 (1.2%)	31/2788 (1.1%)	1.07 (0.66–1.74)	.78	.82
** Composite outcome**	318/2852 (11.2%)	360/2788 (12.9%)	0.86 (0.75–0.99)	.04	.11
**Influenza cohort—age 18–47**					
** All-cause mortality**	<11	<11	1.02 (0.02–51.51)	1.00	1.00
** Hospitalization**	<11	21/558 (3.8%)	**0.20 (0.07–0.56)**	<.001	.005
** Emergency department visit**	34/545 (6.2%)	66/558 (11.8%)	**0.53 (0.35–0.78)**	.001	.006
** ICU admission**	<11	<11	0.26 (0.03–2.28)	.37	.49
** Mechanical ventilation**	<11	<11	3.07 (0.13–75.24)	.49	.57
** Composite outcome**	19/545 (3.5%)	45/558 (8.1%)	**0.43 (0.26–0.73)**	.001	.006
**Influenza cohort—age 48–64**					
** All-cause mortality**	<11	<11	0.25 (0.03–2.22)	.22	.35
** Hospitalization**	39/721 (5.4%)	49/716 (6.8%)	0.79 (0.53–1.19)	.26	.39
** Emergency department visit**	81/721 (11.2%)	96/716 (13.4%)	0.84 (0.64–1.11)	.21	.35
** ICU admission**	12/721 (1.7%)	16/716 (2.2%)	0.74 (0.35–1.56)	.43	.54
** Mechanical ventilation**	<11	<11	0.71 (0.23–2.22)	.55	.60
** Composite outcome**	68/721 (9.4%)	83/716 (11.6%)	0.81 (0.60–1.10)	.18	.33
**Influenza cohort—age 65+**					
** All-cause mortality**	<11	<11	0.30 (0.08–1.07)	.05	.12
** Hospitalization**	82/654 (12.5%)	114/646 (17.6%)	**0.71 (0.55–0.92)**	.01	.04
** Emergency department visit**	121/654 (18.5%)	161/646 (24.9%)	**0.74 (0.60–0.92)**	.005	.02
** ICU admission**	24/654 (3.7%)	32/646 (5.0%)	0.74 (0.44–1.24)	.25	.39
** Mechanical ventilation**	<11	11/646 (1.7%)	0.72 (0.29–1.77)	.47	.57
** Composite outcome**	111/654 (17.0%)	153/646 (23.7%)	**0.72 (0.58–0.89)**	.003	.01

Risk ratios with Benjamini-Hochberg adjusted *P* < .05 are shown in bold.

### Semaglutide-associated risk reduction is preserved across corticosteroid use-strata

To evaluate whether the observed associations were modified by corticosteroid use, the matched cohorts were stratified by documented corticosteroid use within ±7 days of infection (Supplementary Fig. 2). Corticosteroid use within ±7 days of infection was identified in 542 of 7092 (7.6%) semaglutide-treated and 570 of 7092 (8.0%) comparator patients in the matched COVID-19 cohort, and in 133 of 1920 (6.9%) semaglutide-treated and 136 of 1920 (7.1%) comparator patients in the matched influenza cohort. Among COVID-19 patients, semaglutide use was associated with reduced composite outcome risk in both patients without corticosteroid use (RR 0.77, 95% CI 0.68–0.87; *P* < .001) and patients with corticosteroid use (RR 0.81, 95% CI 0.69–0.96; *P* = .01). In the influenza cohort, the composite outcome was reduced in non-users (RR 0.68, 95% CI 0.56–0.83; *P* < .001), with a directionally consistent but non-significant association in users (RR 0.81, 95% CI 0.59–1.12). These results indicate that the association of semaglutide was preserved across patients with or without corticosteroid use at infection.

### Comparisons by therapeutic exposures and vaccination status at infection during the COVID-19 pandemic era

Among patients receiving standard-of-care therapeutics during the COVID-19 pandemic era (defined as infection occurring before May 11, 2023), semaglutide use remained associated with reduced 30-day outcomes (Table 4). Among patients initiating systemic corticosteroids within ±7 days of infection (*n* = 384 vs 408), semaglutide users had a lower composite outcome rate (27.3% vs 36.3%; RR 0.75, 95% CI 0.61–0.93). Among patients receiving Paxlovid antiviral therapy within ±7 days of infection (*n* = 1183 vs 1299), semaglutide users had a lower composite outcome rate (9.6% vs 12.7%; RR 0.76, 95% CI 0.61–0.95). The association was also preserved within pandemic-era vaccination strata: unvaccinated (*n* = 2521 vs 2849; composite RR 0.76, 95% CI 0.65–0.90; *P* < .001) and vaccinated (*n* = 1007 vs 1085; composite RR 0.85, 95% CI 0.66–1.09, not significant), with the stronger association in the higher-risk unvaccinated patients.

**Table 4:** Thirty-day COVID-19 outcomes during the pandemic era among propensity-matched semaglutide users versus metformin comparators, stratified by use of specific co-interventions or by vaccination status. (A) Among patients who initiated systemic corticosteroids within ±7 days of COVID-19 index infection. (B) Among patients who initiated Paxlovid (nirmatrelvir/ritonavir) within ±7 days of COVID-19 index infection. (C) Stratified by vaccination status documented within the 12 months prior to COVID-19 index infection.

Outcome	Semaglutide *n*/*N* (%)	Metformin *n*/*N* (%)	RR (95% CI)	*P*	Adj. *P* (BH-FDR)
**A. Corticosteroids—initiated within ±7 days of index infection (*n* = 384 semaglutide/408 comparator)**					
** All-cause mortality**	<11	22/408 (5.4%)	**0.43 (0.20–0.93)**	.03	.04
** Hospitalization**	83/384 (21.6%)	121/408 (29.7%)	**0.73 (0.57–0.93)**	.010	.03
** Emergency department visit**	111/384 (28.9%)	148/408 (36.3%)	**0.80 (0.65–0.98)**	.03	.04
** Intensive care unit admission**	22/384 (5.7%)	34/408 (8.3%)	0.69 (0.41–1.15)	.15	.18
** Mechanical ventilation**	18/384 (4.7%)	26/408 (6.4%)	0.74 (0.41–1.32)	.30	.30
** Composite outcome**	105/384 (27.3%)	148/408 (36.3%)	**0.75 (0.61–0.93)**	.007	.03
**B. Paxlovid—initiated within ±7 days of index infection (*n* = 1183 semaglutide/1299 comparator)**					
** All-cause mortality**	<11	16/1299 (1.2%)	0.34 (0.13–0.93)	.03	.06
** Hospitalization**	89/1183 (7.5%)	130/1299 (10.0%)	0.75 (0.58–0.97)	.03	.06
** Emergency department visit**	133/1183 (11.2%)	174/1299 (13.4%)	0.84 (0.68–1.04)	.10	.16
** Intensive care unit admission**	24/1183 (2.0%)	36/1299 (2.8%)	0.73 (0.44–1.22)	.23	.23
** Mechanical ventilation**	13/1183 (1.1%)	22/1299 (1.7%)	0.65 (0.33–1.28)	.21	.23
** Composite outcome**	114/1183 (9.6%)	165/1299 (12.7%)	0.76 (0.61–0.95)	.02	.06
**C. COVID-19 vaccination status (within 12 months prior to index infection)**					
** Vaccinated (*n* = 1007 semaglutide/1085 comparator)**					
** All-cause mortality**	<11	<11	0.22 (0.01–4.48)	.50	.55
** Hospitalization**	42/1007 (4.2%)	60/1085 (5.5%)	0.75 (0.51–1.11)	.15	.30
** Emergency department visit**	99/1007 (9.8%)	120/1085 (11.1%)	0.89 (0.69–1.14)	.36	.43
** Intensive care unit admission**	<11	12/1085 (1.1%)	0.81 (0.34–1.91)	.63	.63
** Mechanical ventilation**	<11	<11	0.60 (0.20–1.78)	.35	.43
** Composite outcome**	96/1007 (9.5%)	122/1085 (11.2%)	0.85 (0.66–1.09)	.20	.34
** Unvaccinated (*n* = 2521 semaglutide/2849 comparator)**					
** All-cause mortality**	16/2521 (0.6%)	43/2849 (1.5%)	**0.42 (0.24–0.74)**	.002	.01
** Hospitalization**	164/2521 (6.5%)	236/2849 (8.3%)	0.79 (0.65–0.95)	.01	.05
** Emergency department visit**	279/2521 (11.1%)	355/2849 (12.5%)	0.89 (0.77–1.03)	.11	.27
** Intensive care unit admission**	43/2521 (1.7%)	74/2849 (2.6%)	0.66 (0.45–0.95)	.03	.08
** Mechanical ventilation**	24/2521 (1.0%)	35/2849 (1.2%)	0.77 (0.46–1.30)	.33	.43
** Composite outcome**	216/2521 (8.6%)	320/2849 (11.2%)	**0.76 (0.65–0.90)**	.001	.01

Risk ratios with Benjamini-Hochberg adjusted *P* < .05 are shown in bold.

### Semaglutide-associated risk reduction is consistent across pandemic and seasonal COVID-19 eras

When stratified by COVID-19 era, the association between semaglutide use and 30-day clinical outcomes was observed in both the pandemic phase and the seasonal phase (infections on or after May 11, 2023) (Supplementary Fig. 3). The largest effect on mortality was observed in the pandemic era (RR 0.40, 95% CI 0.22–0.70), where baseline event rates were higher. In the seasonal era, mortality was lower in the semaglutide cohort but did not reach statistical significance (RR 0.48, 95% CI 0.18–1.31). The composite outcome was significantly reduced in both the pandemic era (8.8% vs 11.2%; RR 0.79, 95% CI 0.69–0.90) and the seasonal era (7.5% vs 9.8%; RR 0.77, 95% CI 0.66–0.90). Mechanical ventilation was directionally lower in the pandemic era (RR 0.73, 95% CI 0.46–1.17) and the seasonal era (RR 0.85, 95% CI 0.47–1.54) but did not reach statistical significance in either. Among the subset of patients with type 2 diabetes, the composite outcome remained significantly lower in both the pandemic era (10.6% vs 12.8%; RR 0.83, 95% CI 0.72–0.95) and the seasonal era (8.9% vs 11.8%; RR 0.76, 95% CI 0.64–0.90). These results indicate that the association between semaglutide and 30-day clinical outcomes persists across the major phases of the pandemic transition.

### Within-cohort Sub-analyses: weight loss and dose

**Figure 5:**
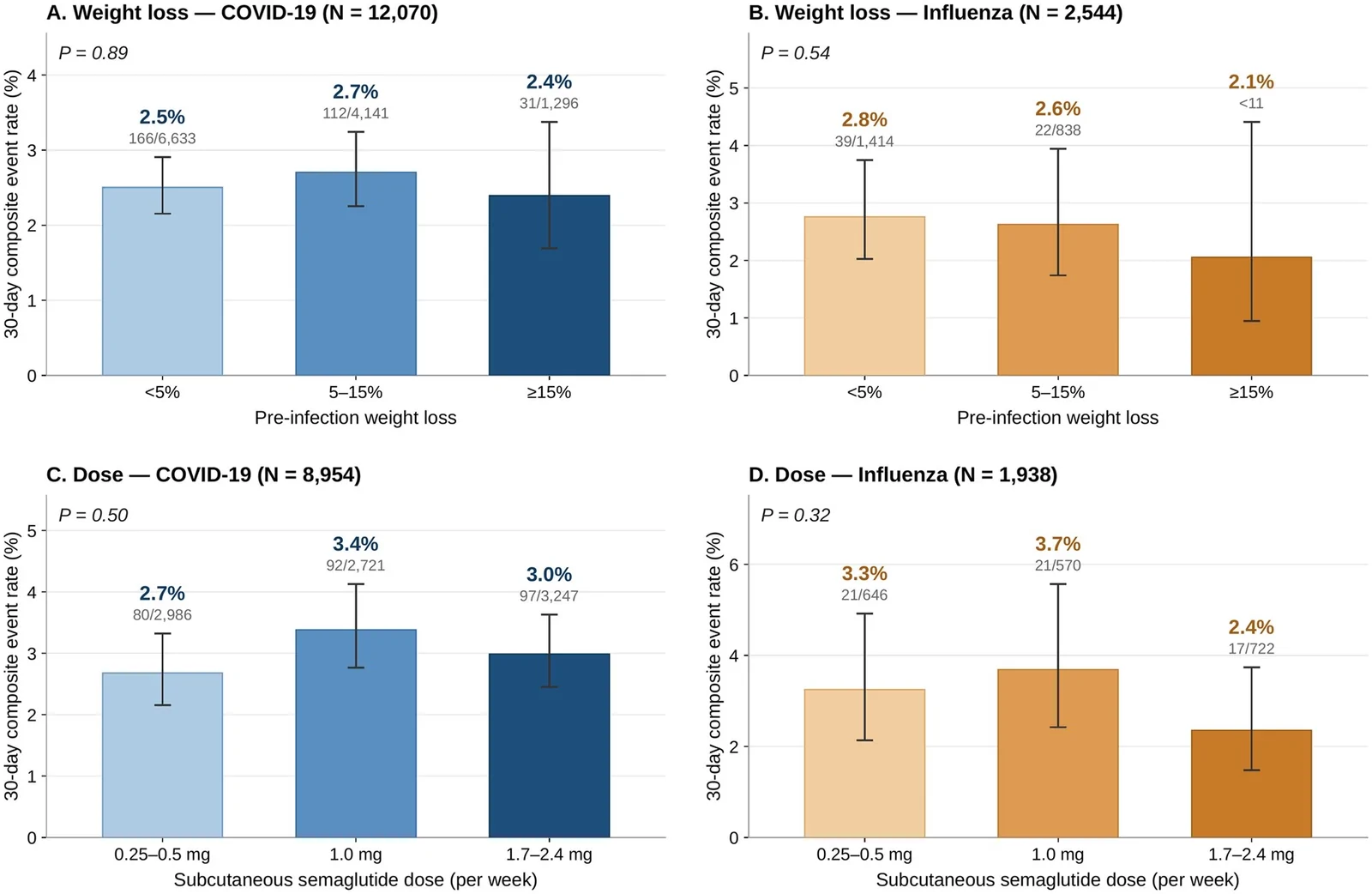
Thirty-day composite outcome rate in semaglutide-treated patients, stratified by pre-infection weight loss (panels A and B) and semaglutide dose (panels C and D). Bar charts showing the rate of the 30-day composite outcome (death, hospitalization, ICU admission, or mechanical ventilation) within the pre-matched semaglutide cohorts. Panels (A) and (B) show event rates by percent weight loss prior to infection in the COVID-19 and influenza cohorts, respectively. Percent weight loss was calculated as the difference between the weight closest to first semaglutide order or administration (within 90 days prior) and the weight closest to the infection date (within 90 days prior), expressed as a percentage of the baseline weight; strata were defined as <5%, 5–15%, and ≥15%. Panels (C) and (D) show event rates by semaglutide dose in the COVID-19 and influenza cohorts, respectively. Dose was determined using the subcutaneous semaglutide dose received closest to the infection date within the 12-month baseline period prior to infection; strata were defined as 0.25–0.5 mg/week (titration), 1.0 mg/week (mid-titration), and 1.7–2.4 mg/week (therapeutic maintenance). Patients without the requisite weight or dose measurements were excluded from the corresponding analysis. Bars indicate the within-stratum event rate and error bars represent 95% Wilson score confidence intervals. *P* values are from the Cochran-Armitage test for trend across the three ordered strata.

Within the semaglutide-treated cohort, neither pre-infection percent weight loss nor maintenance subcutaneous dose was significantly associated with the 30-day composite outcome rate. In the COVID-19 cohort (*n* = 12 070), composite outcome rates by weight loss groups were 2.5% for <5%, 2.7% for 5%–15%, and 2.4% for ≥15% (*P* = .89) (Fig. 5A). In the influenza cohort (*n* = 2544), event rates were 2.8%, 2.6%, and 2.1% across the same weight loss groups (*P* = .54) (Fig. 5B). Stratification by maintenance subcutaneous dose, restricted to patients receiving Ozempic or Wegovy and excluding oral semaglutide (Rybelsus), produced similar results: in the COVID-19 cohort (*n* = 8954), event rates were 2.7% for 0.25–0.5 mg/week, 3.4% for 1.0 mg/week, and 3.0% for 1.7–2.4 mg/week (*P* = .50) (Fig. 5C); in the influenza cohort (*n* = 1938), event rates were 3.3%, 3.7%, and 2.4% across the same dose groups (*P* = .32) (Fig. 5D). Neither higher doses nor greater weight loss were associated with larger reductions in outcomes, suggesting the association may reflect drug-specific effects.

### Negative control outcome analysis supports minimal residual confounding

To assess the potential for residual confounding, we evaluated a negative control outcome, 30-day incident urinary tract infection, which has no plausible causal relationship with pre-infection semaglutide exposure but is subject to ascertainment and confounding similar to the primary outcomes. Urinary tract infection occurred in 33 of 7092 (0.5%) semaglutide-treated and 29 of 7092 (0.4%) comparator patients in the COVID-19 cohort (RR 1.14, 95% CI 0.69–1.87; *P* = .61), and in <11 of 1920 semaglutide-treated and <11 of 1920 comparator patients in the influenza cohort (RR 1.12, 95% CI 0.43–2.91; *P* = .81). The absence of an association in either cohort argues against substantial residual confounding or differential surveillance as an explanation for the observed differences in outcomes (Supplementary Table 2).

## Discussion

In this large multi-site real-world cohort study, prior exposure to semaglutide was associated with significantly reduced 30-day risk of severe outcomes following both COVID-19 and influenza infection. The associations included a 61% relative reduction in 30-day mortality for COVID-19 and a 71% relative reduction for influenza, while reductions in intensive care admission and mechanical ventilation were directionally consistent but did not reach statistical significance against the metformin comparator. The semaglutide-associated reduction in the composite severity outcome was similar across vaccinated and unvaccinated patients and persisted across both the pandemic and seasonal phases of COVID-19. Among organ-specific outcomes, semaglutide was associated with significantly lower acute kidney injury in both cohorts, whereas cardiac and pulmonary associations were directionally consistent but did not reach statistical significance.

This study adds to existing observational evidence suggesting that GLP-1 receptor agonists may favorably influence outcomes during acute illness and respiratory infections [12, 37], and it advances the field in several ways. The cohort spans both the pandemic and post-pandemic periods, includes two distinct respiratory viruses, and applies a strict propensity-score matching approach. The parallel evaluation in influenza is an important biological control: the consistency of effect direction across two distinct respiratory viruses argues against narrow virus-specific mechanisms and instead is compatible with a host-directed effect [3, 4].

Previous work provides plausible biological rationale for a favorable association of semaglutide against severe disease following viral infection. GLP-1 receptor activation exerts effects beyond glycemic control, including reductions in systemic inflammation, modulation of macrophage and T-cell activity, improvement in endothelial function, and reductions in adverse cardiovascular events [8–10]. During acute respiratory viral infection, dysregulated host inflammation, endothelial injury, and microvascular thrombosis are central drivers of severe disease [2, 5]. A pharmacotherapy that attenuates these pathways could plausibly be associated with lower risk of decompensation following viral exposure; these mechanisms are hypotheses, and no mechanistic or biomarker data were measured in this study.

The within-cohort sub-analyses indicated that neither pre-infection percent weight loss nor maintenance subcutaneous dose was significantly associated with the 30-day composite outcome rate. These exploratory analyses did not establish that the observed association is independent of weight loss or dose, given subset sample sizes, substantial missing data, and the absence of formal interaction testing. However, the findings do raise the possibility of pleiotropic effects on factors such as immune and vascular function inflammation at sub-maximal therapeutic exposure. This finding is consistent with the cardiovascular benefits that have been observed with semaglutide, which appear to be at least partially independent of weight loss magnitude [8, 9, 38].

There are several limitations to this study. First, despite propensity-score matching, residual confounding by unmeasured variables remains possible, including duration of diabetes, insulin use, severity of chronic kidney and cardiovascular disease, socioeconomic status, insurance type, frailty, healthcare engagement, and adherence behaviors not fully captured by electronic health record data. The use of a metformin active comparator, matched on HbA1c, BMI, healthcare utilization, and major comorbidities, is expected to reduce but not eliminate confounding by these factors, because metformin-treated patients share a diabetes diagnosis, comparable metabolic management, and similar healthcare access with semaglutide-treated patients. In addition, a negative control outcome analysis using 30-day urinary tract infection showed no association in either cohort, providing empirical support that substantial residual confounding is unlikely (Supplementary Table 2). Second, ascertainment of vaccination, antiviral use, and prior infection may be incomplete if they occurred outside the health system. The substantially higher documented antiviral use compared with documented vaccination in both cohorts likely reflects this differential ascertainment, since antivirals are typically prescribed at the same encounter as the positive infection test and captured directly in the EHR, whereas vaccinations are commonly administered at pharmacies, employer clinics, or community sites outside the network. As a result, the unvaccinated stratum likely includes a meaningful proportion of patients who were vaccinated outside the participating health systems. Third, the study evaluated outcomes within 30 days of infection, and longer-term outcomes including post-acute sequelae and cardiovascular events beyond this window were not assessed. Beyond 30 days, attributing events to the viral infection becomes difficult given the high baseline rates of cardiovascular, renal, and metabolic conditions expected in a cohort enriched for diabetes and obesity. Fourth, dose and weight-loss measurements were not available for the entire semaglutide cohort, which reduced sample size and limited statistical power to detect modest differences across groups in these sub-analyses. Fifth, although the analysis used data from a federated network of academic medical centers, between-site variation in care patterns and practices may contribute to residual heterogeneity. Sixth, although the analysis used metformin as an active comparator to reduce healthy-user and healthcare-engagement biases. However, metformin is itself pharmacologically active, so the contrast estimates the effect of semaglutide relative to metformin rather than relative to no treatment. Comparisons against other GLP-1 receptor agonists, SGLT2 inhibitors, or DPP-4 inhibitors could help to further isolate semaglutide-specific effects.

In summary, relative to a metformin active comparator, prior semaglutide exposure was associated with lower 30-day mortality, hospitalization, and composite clinical outcomes following both COVID-19 and influenza infection in a large propensity-matched real-world cohort. Reductions in intensive care admission and mechanical ventilation were directionally consistent but did not reach statistical significance, and among organ-specific outcomes acute kidney injury was significantly lower in both cohorts. The associations were consistent across vaccination status and COVID-19 eras. Because the study is observational, these findings should be interpreted as associations rather than evidence of benefit. These findings support continued investigation of GLP-1 receptor agonists as potential modifiers of respiratory viral disease severity, with prospective randomized evaluation and active-comparator observational analyses representing the most informative next steps.

## Supplementary Material

bpag042_Supplementary_Data

## References

[1] GBD 2023 Lower Respiratory Infections and Antimicrobial Resistance Collaborators. Global burden of lower respiratory infections and aetiologies, 1990-2023: a systematic analysis for the Global Burden of Disease Study 2023. Lancet Infect Dis 2026;26:343–61. 10.1016/S1473-3099(25)00689-9 41412141

[2] Williamson EJ , Walker AJ, Bhaskaran K et al Factors associated with COVID-19-related death using OpenSAFELY. Nature 2020;584:430–6. 10.1038/s41586-020-2521-4 PMC7611074 32640463

[3] Apicella M , Campopiano MC, Mantuano M et al COVID-19 in people with diabetes: understanding the reasons for worse outcomes. Lancet Diabetes Endocrinol 2020;8:782–92. 10.1016/S2213-8587(20)30238-2 PMC7367664 32687793

[4] Honce R , Schultz-Cherry S. Impact of obesity on influenza A virus pathogenesis, immune response, and evolution. Front Immunol 2019;10:1071. 10.3389/fimmu.2019.01071 PMC6523028 31134099

[5] Groff D , Sun A, Ssentongo AE et al Short-term and long-term rates of postacute sequelae of SARS-CoV-2 infection: a systematic review. JAMA Netw Open 2021;4:e2128568. 10.1001/jamanetworkopen.2021.28568 PMC8515212 34643720

[6] Darweesh M , Mohammadi S, Rahmati M et al Metabolic reprogramming in viral infections: the interplay of glucose metabolism and immune responses. Front Immunol 2025;16:1578202. 10.3389/fimmu.2025.1578202 PMC12122472 40453076

[7] Moiz A , Filion KB, Tsoukas MA et al The expanding role of GLP-1 receptor agonists: a narrative review of current evidence and future directions. EClinicalMedicine 2025;86:103363. 10.1016/j.eclinm.2025.103363 PMC12303005 40727007

[8] Marso SP , Bain SC, Consoli A et al; SUSTAIN-6 Investigators. Semaglutide and cardiovascular outcomes in patients with type 2 diabetes. N Engl J Med 2016;375:1834–44. 10.1056/NEJMoa1607141 27633186

[9] Lincoff AM , Brown-Frandsen K, Colhoun HM et al; SELECT Trial Investigators. Semaglutide and cardiovascular outcomes in obesity without diabetes. N Engl J Med 2023;389:2221–32. 10.1056/NEJMoa2307563 37952131

[10] Drucker DJ. Mechanisms of action and therapeutic application of glucagon-like peptide-1. Cell Metab 2018;27:740–56. 10.1016/j.cmet.2018.03.001 29617641

[11] Greco S , Monda VM, Valpiani G et al The impact of GLP-1 RAs and DPP-4is on hospitalisation and mortality in the COVID-19 era: a two-year observational study. Biomedicines 2023;11:2292. 10.3390/biomedicines11082292 PMC10452157 37626788

[12] Kahkoska AR , Abrahamsen TJ, Alexander GC et al; N3C Consortium. Association between glucagon-like peptide 1 receptor agonist and sodium-glucose cotransporter 2 inhibitor use and COVID-19 outcomes. Diabetes Care 2021;44:1564–72. 10.2337/dc21-0065 PMC8323175 34135013

[13] Nyland JE , Raja-Khan NT, Bettermann K et al Diabetes, drug treatment, and mortality in COVID-19: a Multinational Retrospective Cohort Study. Diabetes 2021;70:2903–16. 10.2337/db21-0385 PMC8660979 34580086

[14] Israelsen SB , Pottegård A, Sandholdt H et al Comparable COVID-19 outcomes with current use of GLP-1 receptor agonists, DPP-4 inhibitors or SGLT-2 inhibitors among patients with diabetes who tested positive for SARS-CoV-2. Diabetes Obes Metab 2021;23:1397–401. 10.1111/dom.14329 PMC8014019 33502076

[15] Khunti K , Knighton P, Zaccardi F et al Prescription of glucose-lowering therapies and risk of COVID-19 mortality in people with type 2 diabetes: a nationwide observational study in England. Lancet Diabetes Endocrinol 2021;9:293–303. 10.1016/S2213-8587(21)00050-4 PMC8009618 33798464

[16] Lund JL , Richardson DB, Stürmer T. The active comparator, new user study design in pharmacoepidemiology: historical foundations and contemporary application. Curr Epidemiol Rep 2015;2:221–8. 10.1007/s40471-015-0053-5 PMC4778958 26954351

[17] Yoshida K , Solomon DH, Kim SC. Active-comparator design and new-user design in observational studies. Nat Rev Rheumatol 2015;11:437–41. 10.1038/nrrheum.2015.30 PMC4486631 25800216

[18] Schneeweiss S , Rassen JA, Glynn RJ et al High-dimensional propensity score adjustment in studies of treatment effects using health care claims data. Epidemiology 2009;20:512–22. 10.1097/EDE.0b013e3181a663cc PMC3077219 19487948

[19] Rosenbaum PR , Rubin DB. The central role of the propensity score in observational studies for causal effects. Biometrika 1983;70:41–55. 10.1093/biomet/70.1.41

[20] Austin PC. An introduction to propensity score methods for reducing the effects of confounding in observational studies. Multivariate Behav Res 2011;46:399–424. 10.1080/00273171.2011.568786 PMC3144483 21818162

[21] Austin PC. Optimal caliper widths for propensity-score matching when estimating differences in means and differences in proportions in observational studies. Pharm Stat 2011;10:150–61. 10.1002/pst.433 PMC3120982 20925139

[22] Franklin JM , Patorno E, Desai RJ et al Emulating randomized clinical trials with nonrandomized real-world evidence studies: first results from the RCT DUPLICATE initiative. Circulation 2021;143:1002–13. 10.1161/CIRCULATIONAHA.120.051718 PMC7940583 33327727

[23] Lipsitch M , Tchetgen Tchetgen E, Cohen T. Negative controls: a tool for detecting confounding and bias in observational studies. Epidemiology 2010;21:383–8. 10.1097/EDE.0b013e3181d61eeb PMC3053408 20335814

[24] Wilding JPH , Batterham RL, Calanna S et al STEP 1 Study Group. Once-weekly semaglutide in adults with overweight or obesity. N Engl J Med 2021;384:989–1002. 10.1056/NEJMoa2032183 33567185

[25] Austin PC. Balance diagnostics for comparing the distribution of baseline covariates between treatment groups in propensity-score matched samples. Stat Med 2009;28:3083–107. 10.1002/sim.3697 PMC3472075 19757444

[26] Katz D , Baptista J, Azen SP, Pike MC. Obtaining confidence intervals for the risk ratio in cohort studies. Biometrics 1978;34:469–74. 10.2307/2530610

[27] Agresti A , Kateri M. Categorical data analysis. In: International Encyclopedia of Statistical Science. Berlin, Heidelberg: Springer, 2025, 408–11.

[28] Newcombe RG. Two-sided confidence intervals for the single proportion: comparison of seven methods. Stat Med 1998;17:857–72. 10.1002/(SICI)1097-0258(19980430)17:8<857::AID-SIM777>3.0.CO;2-E 9595616

[29] Cochran WG. Some methods for strengthening the common χ^2^ tests. Bioethics 1954;10:417. 10.2307/3001616

[30] Armitage P. Tests for linear trends in proportions and frequencies. Biometrics 1955;11:375–86. 10.2307/3001775

[31] Benjamini Y , Hochberg Y. Controlling the false discovery rate: a practical and powerful approach to multiple testing. J R Stat Soc 1995;57:289–300. 10.1111/j.2517-6161.1995.tb02031.x

[32] Wang SV , Pinheiro S, Hua W et al STaRT-RWE: structured template for planning and reporting on the implementation of real world evidence studies. BMJ 2021;372:m4856. 10.1136/bmj.m4856 PMC8489282 33436424

[33] Langan SM , Schmidt SA, Wing K et al The reporting of studies conducted using observational routinely collected health data statement for pharmacoepidemiology (RECORD-PE). BMJ 2018;363:k3532. 10.1136/bmj.k3532 PMC6234471 30429167

[34] Wang SV , Schneeweiss S, Berger ML et al; Joint ISPE-ISPOR Special Task Force on Real World Evidence in Health Care Decision Making. Reporting to improve reproducibility and facilitate validity assessment for healthcare database studies V1.0. Pharmacoepidemiol Drug Saf 2017;26:1018–32. 10.1002/pds.4295 PMC5639362 28913963

[35] Murugadoss K , Rajasekharan A, Malin B et al Building a best-in-class automated de-identification tool for electronic health records through ensemble learning. Patterns (N Y) 2021;2:100255. 10.1016/j.patter.2021.100255 PMC8212138 34179842

[36] Venkatakrishnan AJ et al Clinical nSights: a software platform to accelerate real world oncology analyses. J Clin Oncol 2024.

[37] Han S , Liu Y, Xing B et al Association of glucagon-like peptide-1 receptor agonist use with risk of infections: a systematic review and meta-analysis. J Infect 2025;91:106645. 10.1016/j.jinf.2025.106645 41173399

[38] Deanfield J , Lincoff AM, Kahn SE et al Semaglutide and cardiovascular outcomes by baseline and changes in adiposity measurements: a prespecified analysis of the SELECT trial. Lancet 2025;406:2257–68. 10.1016/S0140-6736(25)01375-3 41138739

