# Supplementary figures and images for: Semaglutide use linked to lower COVID-19 and influenza severity with better renal outcomes after pandemic or seasonal infection
Source: Biol Methods Protoc. 2026 Jul 17;11(1):bpag042. doi: 10.1093/biomethods/bpag042 (PMC13425107; doi:10.1093/biomethods/bpag042)

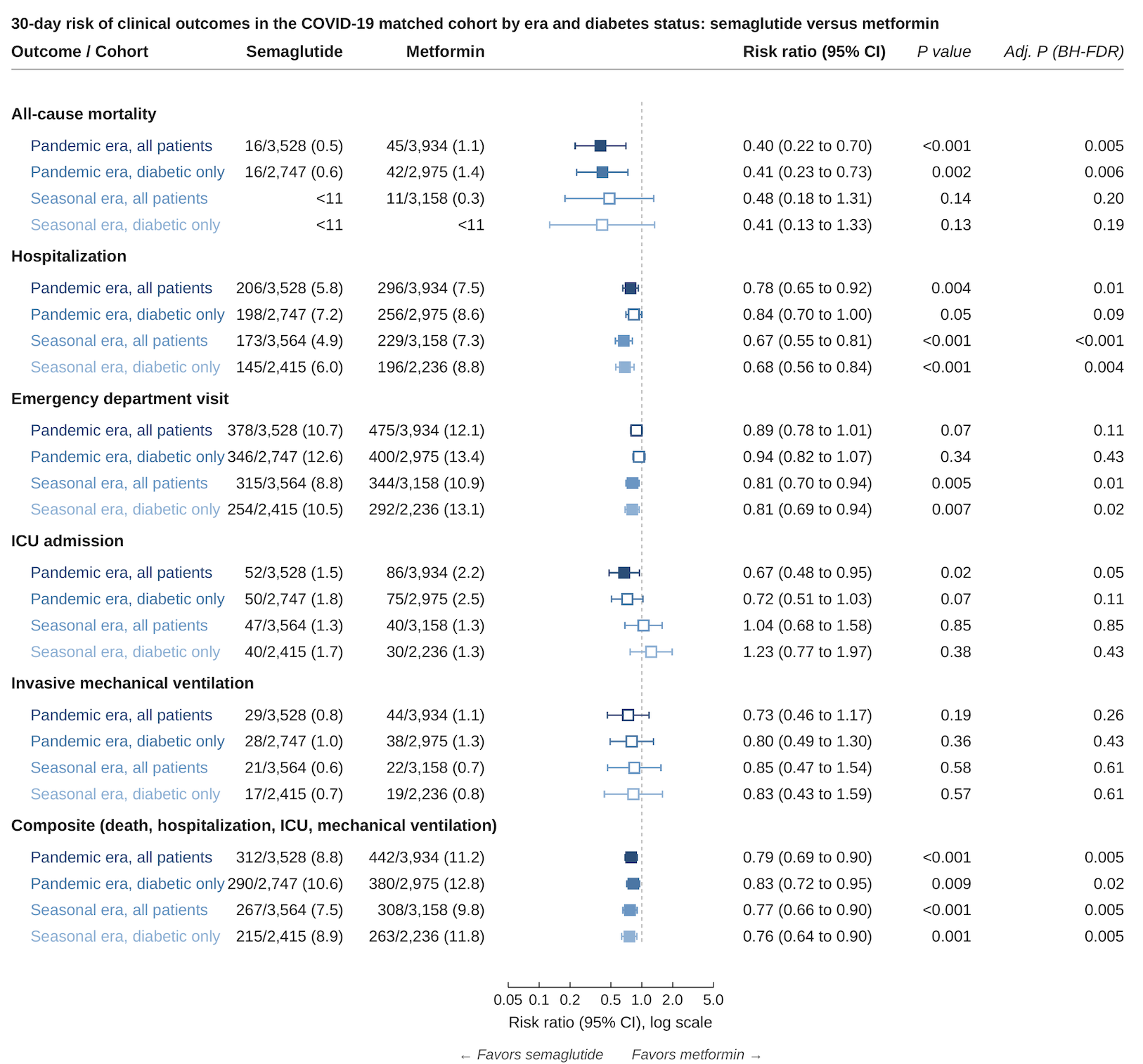

Supplement: bpag042_Supplementary_Data [file bpag042_supplementary_data.zip › Screenshot 2026-07-10 at 6.27.36 PM.png]

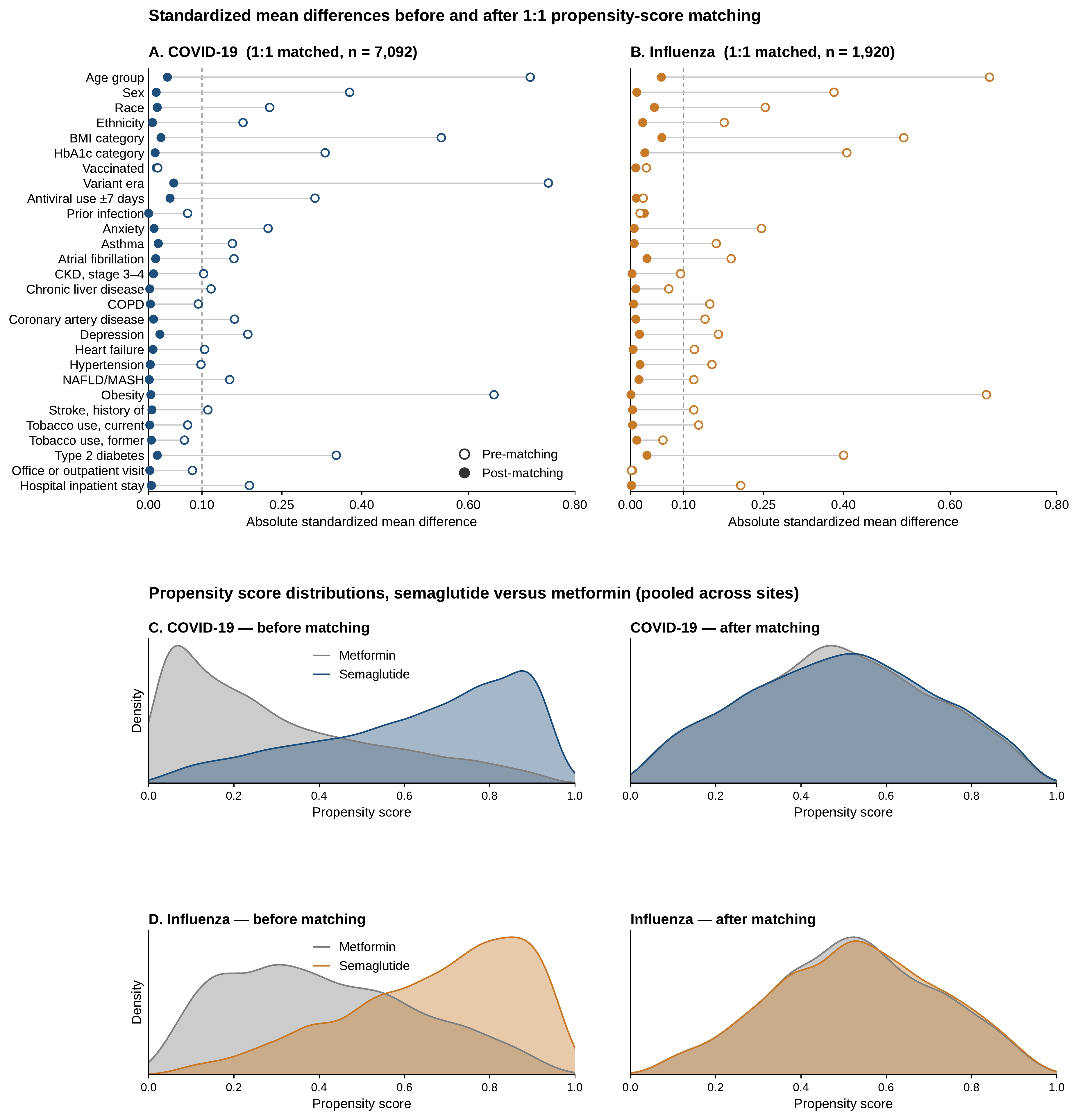

Supplement: bpag042_Supplementary_Data [file bpag042_supplementary_data.zip › Figure_S1.tif]

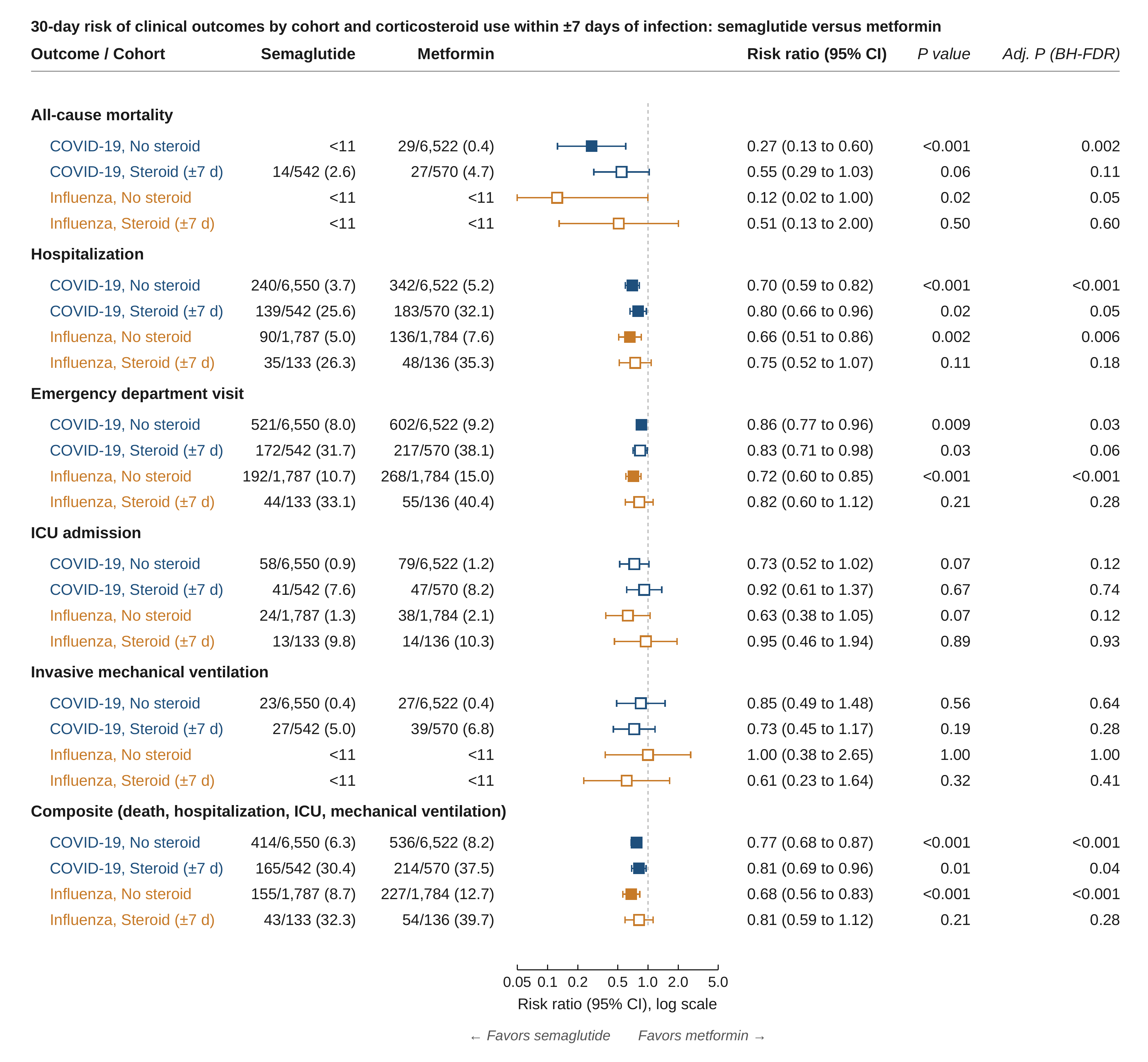

Supplement: bpag042_Supplementary_Data [file bpag042_supplementary_data.zip › Figure_S2.tif]
